# Probing long-range carrier-pair spin–spin interactions in a conjugated polymer by detuning of electrically detected spin beating

**DOI:** 10.1038/ncomms7688

**Published:** 2015-04-14

**Authors:** Kipp J. van Schooten, Douglas L. Baird, Mark E. Limes, John M. Lupton, Christoph Boehme

**Affiliations:** 1 Department of Physics and Astronomy, University of Utah, 115 South 1400 East, Salt Lake City, Utah 84112-0830, USA; 2 Institute of Experimental and Applied Physics, University of Regensburg, Universitätsstrasse 31, 93053 Regensburg, Germany

## Abstract

Weakly coupled electron spin pairs that experience weak spin–orbit interaction can control electronic transitions in molecular and solid-state systems. Known to determine radical pair reactions, they have been invoked to explain phenomena ranging from avian magnetoreception to spin-dependent charge-carrier recombination and transport. Spin pairs exhibit persistent spin coherence, allowing minute magnetic fields to perturb spin precession and thus recombination rates and photoreaction yields, giving rise to a range of magneto-optoelectronic effects in devices. Little is known, however, about interparticle magnetic interactions within such pairs. Here we present pulsed electrically detected electron spin resonance experiments on poly(styrene-sulfonate)-doped poly(3,4-ethylenedioxythiophene) (PEDOT:PSS) devices, which show how interparticle spin–spin interactions (magnetic-dipolar and spin-exchange) between charge-carrier spin pairs can be probed through the detuning of spin-Rabi oscillations. The deviation from uncoupled precession frequencies quantifies both the exchange (<30 neV) and dipolar (23.5±1.5 neV) interaction energies responsible for the pair's zero-field splitting, implying quantum mechanical entanglement of charge-carrier spins over distances of 2.1±0.1 nm.

## Abstract (web summary)

Interactions between weakly coupled pairs of electron spins are thought to play a role in biological magnetoreception and spin-dependent carrier dynamics in semiconductors. Here, the authors investigate such intrapair dipolar and exchange interactions in a polymer using electrically detected magnetic resonance experiments.

## Graphical abstract

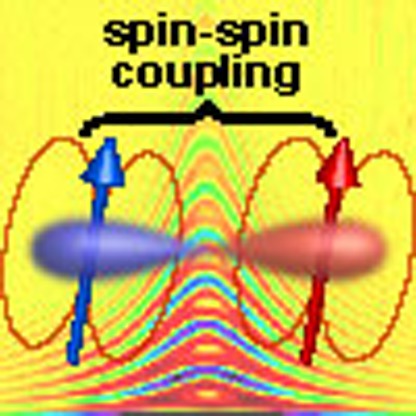

When Erwin Schrödinger conceived his famous *Gedankenexperiment* on the non-deterministic quantum mechanical nature of cats, he may have intuited that quantum superpositions, entanglement and oscillations between eigenstates are fundamental to many aspects of life. Indeed, magnetoreception in several avian species^1^ and insects^2^ has been proposed to arise due to photoinduced radical pair formation, where spin permutation symmetry becomes more sensitive to external magnetic fields the longer the radical spins preserve their phase coherence^3^. Ultimately, the pairs recombine to trigger a biochemical reaction, where the recombination rate is thought to be spin dependent. Room-temperature coherence of spin pairs on the timescale of hundreds of microseconds has been invoked to explain extraordinary magnetosensitivity on the microtesla scale^3^,^4^. The nature of the underlying radical pair mechanism^5^ can be tested by a variety of experiments, the most elegant being the electromagnetically induced disorientation of birds as they are exposed to radiofrequency waves resonant with the radical pair Zeeman splitting induced by Earth's magnetic field^1^.

Yet while impressive progress has been made in studying the radical pair mechanism in reaction yields of natural and biomimetic solution-based systems^6^, an intriguing corollary offers itself in terms of spin-dependent electron–hole recombination in organic semiconductors^7^. In contrast to solution-based systems, spin coherence phenomena can be read out electrically, in miniscule volumes of materials, which enhances the magnetic field homogeneity in spin resonance spectroscopy. Electrically detected pulsed electron spin resonance (ESR) can be carried out at arbitrary Zeeman splittings (*B*_0_ field strengths), provided the strength of local hyperfine fields is exceeded^8^, a condition not strictly met under theories for magnetoreception in Earth's field. For charge-carrier spin pairs in organic semiconductors, this approach has revealed coherent spin dephasing times of order 1 μs at room temperature in spin-echo experiments^9^, coherent spin-Rabi oscillations in the device current^7^ and even coherent spin beating when both pair partners are in resonance with the driving electromagnetic field^10^. The crucial aspect of the radical pair process which has not been addressed yet relates to the magnetic interaction of one spin species with the other: how does the magnetic field of spin a influence the precession of spin b in an external magnetic field (see Fig. 1 for detailed discussion)? This correction to spin precession, in effect a zero-field splitting term, arises due to both dipolar and exchange interactions, which decrease strongly with carrier separation. Crucially, this energetic splitting places a lower limit on the spatial extension of the entangled spin state—the size of ‘Schrödinger's cat' or, for the pair systems considered, the distance of the pair partners. Dipolar coupling lifts the degeneracy of singlet and triplet spin configurations, and has recently even been demonstrated to be detectable on the micrometre length scale in trapped ion systems^11^, where spin coherence times can exceed seconds.

This study shows that the simple device current of a polymer diode can contain such information on spin entanglement of site-separated spins. We employ the widely used conducting polymer PEDOT:PSS as the active material due to its comparatively low density of hydrogen nuclei from which carriers are shielded effectively. At low temperatures, when the acceptor sites bind mobile holes, conduction gives way to a semiconducting state possessing conventional diode-like rather than Ohmic current–voltage characteristics. We use very short (ns range), powerful (kW range) coherent microwave pulses to coherently drive magnetic resonant spin-Rabi nutation in the carrier-pair spin manifold and monitor the resulting oscillation of free charge-carrier density in the device. Such an experiment is known as pulsed electrically detected magnetic resonance (pEDMR)^12^,^13^. Spin-beat oscillations of the Rabi nutations of the two charge carriers develop as each partner of the coupled pair is coherently but differently driven in the resonant electromagnetic field. This beat oscillation leads to an effective doubling in precession frequency. The spin–spin interaction strength due to exchange and magnetic-dipolar coupling becomes quantifiable by measuring the precise variation in Rabi beat frequencies while detuning the system from its resonance condition. The exact quantification of the precession frequency components under detuning has previously been hampered by the significant hyperfine interactions common to organic semiconductors. This limitation necessitates an appropriate choice of material system for unambiguously demonstrating the use of detuned spin-beat Rabi oscillations as a spectroscopic technique to quantify zero-field splitting within the pair. Here we use two methods to constrain the spin–spin interaction strengths: first, through comparison of our measurements with the analytical solution for correlated spin-pair precession in the limit of weak coupling (that is, *J*=*D*=0, with *J* the exchange energy and *D* the dipolar coupling); and second, by comparing the measured deviation from the weak coupling limit with an accurate numerical simulation for finite coupling within the pair. This analysis results in separate constraints for both the exchange (|*J*|<30 neV) and dipolar (|*D*|=23.5±1.5 neV) coupling energies, which contribute to the total spin–spin interaction energy. The mutual coupling of these spins yields a magnetic field correction of order 200 μT (depending on the limit taken on the exchange interaction). Crucially, this zero-field splitting influences the ultimate limit on the sensitivity of radical pair-based magnetometers^8^. By considering the dipolar portion of this splitting alone, a mean intercharge separation of 2.1±0.1 nm is implied, describing the distance over which carrier-pair entanglement persists. Our approach demonstrates the applicability of state-of-the-art spin resonance techniques to provide microscopic quantitative insight into spin-dependent transitions in the conductivity of organic semiconductors. This demonstration provides an expansion of the spectroscopic base of ‘organic spintronics' where conventional spectroscopic tools in spintronics, such as the magneto-optic Kerr effect or inductively detected magnetic resonance spectroscopy, cannot be applied^14^.

## Results

### Electrical detection of coherent spin beating

**Figure 1:**
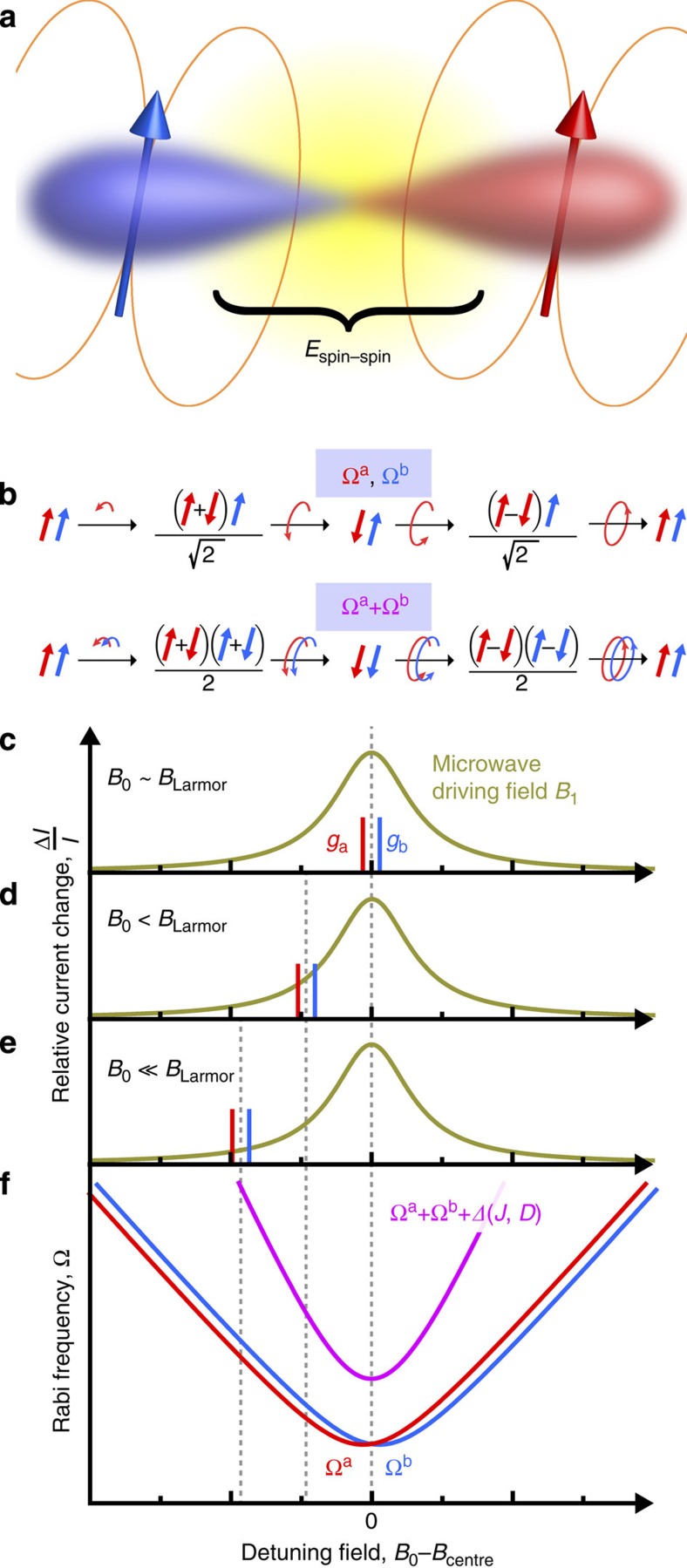
Probing carrier-pair spin–spin interactions by resonantly driving harmonic Rabi oscillations. (**a**) Spin–spin interactions within charge-carrier pairs arise from spin-exchange *J* and magnetic-dipolar coupling *D*. (**b**) Under magnetic resonance conditions, a weak oscillating driving field *B*_1_ forces the spin state of a single charge carrier into precession, while the pair partner at a shifted Larmor frequency is out of resonance and therefore left unperturbed. In contrast, when a strong driving field is applied, which exceeds the pair's average Larmor separation, both carriers oscillate simultaneously (**c**), producing a beat oscillation (the harmonic) within the pair. This spin beating is reflected in the measured current by spin-dependent charge carrier transitions (recombination and dissociation). As the static magnetic field, *B*_0_, which induces Zeeman splitting of the spin states, is detuned away from magnetic resonance of either pair partner (**d**,**e**), both the fundamental and the harmonic of the Rabi oscillation frequencies shift according to equation (1). (**f**) When spin–spin coupling is non-zero, the harmonic of the Rabi frequency acquires an additional shift *Δ* that depends on *J* and *D*.

pEDMR is an ESR-based experiment where only spin resonant transitions are observed which control, directly or indirectly, the conductivity of a material^12^,^13^. As pEDMR is insensitive to magnetic resonance effects of spin states that do not affect conductivity, the current-detected spin-resonance signals on PEDOT:PSS reported here arise exclusively due to spin-dependent processes involving charge carriers. Therefore, since the data discussed in the following shows the characteristic spin motion of weakly coupled spin-½ carrier pairs, the signals must be due to charge-carrier pairs. As in conventional ESR, the quantization axis is defined by a static external magnetic field, *B*_0_, establishing a Zeeman energy eigenbasis. Besides *B*_0_, the spin-pair eigenstates depend on the coupling interactions within the pair (that is, spin-exchange, *J*, and magnetic-dipolar, *D*, as illustrated in Fig. 1a) and local nuclear spin interactions (the magnetic fields generated by the abundant protons, the hyperfine fields). Resonant transitions between the four eigenstates of a spin-½ pair can be induced by an oscillating driving field, *B*_1_, aligned perpendicular to the static field, when the microwave frequency (*ω*_MW_/2*π*≈9.7 GHz for *B*_0_≈340 mT) corresponds to the Zeeman energy term. Although continuous-wave microwave fields are sufficient for driving these transitions^15^,^16^,^17^, the application of pulsed fields provides access to additional information, such as spin and charge relaxation rates^9^,^18^, spin–spin coupling strengths^19^,^20^ and the charge-nuclear spin-hyperfine interaction^10^,^21^. Such coherent state manipulation requires a microwave pulse duration, *τ*, which is shorter than the dephasing time, *T*_2_, of the state being probed, opening possibilities for quantum control of macroscopic observables such as the current, as well as more general applications in quantum-information processing^22^,^23^,^24^.

The observable for such coherent spin manipulation is the total charge, *Q*, arising from carrier pairs occupying a particular eigenstate after a driving time, *τ*, and is acquired by integration of the current transient following excitation (see Methods and ref. 13). A straightforward demonstration of coherent control of eigenstate population is through the continuous driving of a spin transition between two eigenstates, that is, Rabi nutation^7^,^10^,^13^,^23^,^25^,^26^,^27^. The nutation frequency, Ω_R_, of this oscillation depends on the system's spin Hamiltonian^28^,^29^,^30^,^31^ (see Supplementary Note 1) and the magnitude of the driving field, *B*_1_ (refs 10, 13, 21, 25, 26, 27, 32). As the eigenstates are defined, in part, by the magnitudes of *J* and *D*, the general expression for the nutation frequency between eigenstates of the system is sensitive to these spin coupling parameters^28^,^29^,^30^,^31^. We begin by considering the analytical expression for Rabi nutation in the weak coupling limit and in the absence of any hyperfine distribution,


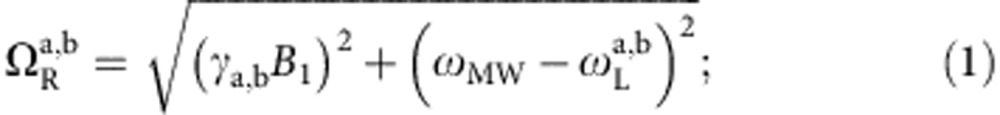


that is, the case when |*J*|+|*D*|≪Δ*ω*_L_, where 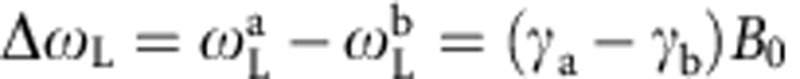 is the difference in Larmor frequency for each charge a, b with γ_a,b_ their respective gyromagnetic ratios, as marked in Fig. 1c (refs 28, 29, 30, 31, 32). For our model system, this is an appropriate condition for the weakly interacting carriers confined to localized sites within PEDOT:PSS^33^. Previous studies have tested the linear dependence of this relation on *B*_1_ field strength in organic light-emitting diodes driven on-resonance (that is, for 
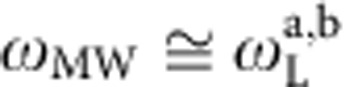
)^7^. Under these conditions, it was confirmed that the fundamental Rabi frequency scales as Ω_R_=γ*B*_1_ when the driving field was selective for a single carrier within a weakly spin-coupled pair^7^. Further, by increasing *B*_1_ such that both carriers of the pair are driven (as described in Fig. 1b), a harmonic Rabi frequency emerges, behaving as 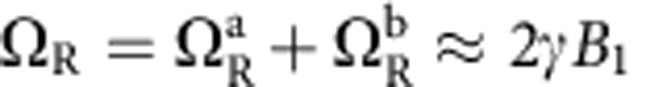 (refs 10, 14, 21, 26). Observation of this harmonic in combination with the fundamental Rabi frequency implies that the magnetic resonance feature observed arises from a weakly coupled pair.

In the following, we probe the effect of detuning *ω*_MW_−*ω*_L_ in equation (1), the result of which is sketched in Fig. 1c–e. The frequencies and intensities of the Rabi oscillation components depend on detuning as determined by the spin Hamiltonian of the charge-carrier pairs and thus on spin coupling within the pairs. Panel (f) depicts the hyperbolic dependence of the Rabi frequency components (equation (1)) as a function of detuning for the case of weak spin–spin coupling. In the presence of finite coupling energies, the linear sum of fundamental Rabi frequencies producing the harmonic is modified by an offset *Δ*, which is a function of *J* and *D*. Unlike the fundamental Rabi oscillation components Ω_a_ and Ω_b_, *Δ* is independent of the signs of J and D. Thus, as *Δ* depends on the magnitudes |*J*| and |*D*|, it serves as a direct observable of the spin–spin interaction energy.

### Material system

**Figure 2:**
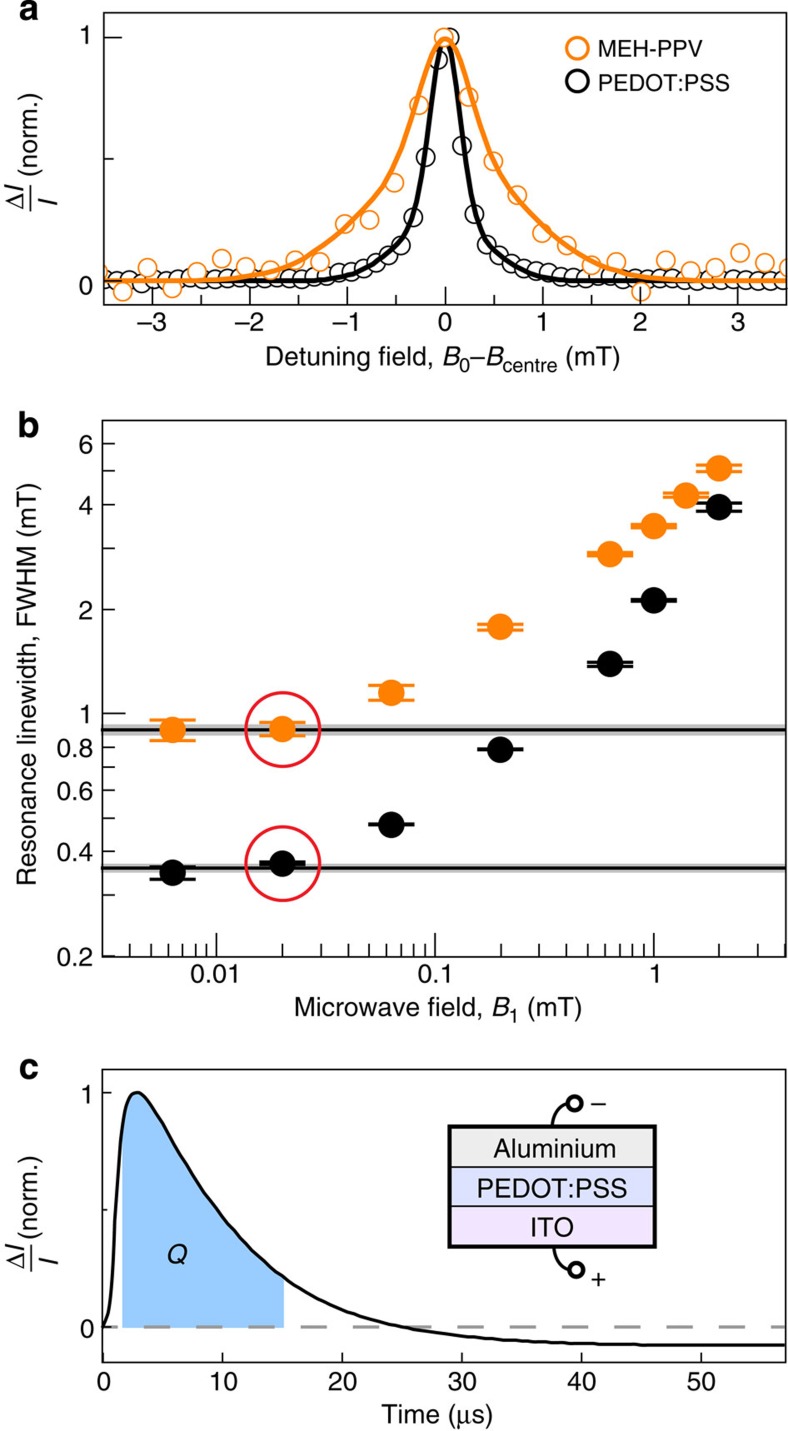
Intrinsic pEDMR spectral linewidth of two conducting polymers with different hyperfine nuclear–electronic coupling strengths. (**a**) pEDMR lineshapes of MEH-PPV (orange) and PEDOT:PSS (black), at 5 K for an applied microwave field with frequency *f*_MW_=9.72691 GHz. PEDOT:PSS has a lower hydrogen content, reducing the effective hyperfine field and narrowing the resonance. (**b**) pEDMR linewidths as a function of microwave driving field, *B*_1_, demonstrating the absence of microwave power broadening for the spectra in **a**. The data points corresponding to the spectra in **a** are indicated by red circles. At larger *B*_1_ fields, power broadening sets in. (**c**) Transient dynamics following resonant excitation. The change in current is proportional to the time evolution of eigenstate density operators during the microwave pulse, with the number of resonant charges monitored by integration of these time dynamics, as discussed in the Methods. This integrated current, *Q*, is recorded as a function of *τ*, *B*_1_ and *B*_0_.

To reveal the detuning of Rabi oscillations, minimal inhomogeneous broadening of the resonance line is crucial. The interaction of charge-carrier spins with local hyperfine fields, *B*_hyp_, primarily generated by hydrogen nuclei, is well documented for organic semiconductors^10^,^21^ and is presumed to be the origin of some of the intriguing magnetic field effects unique to these material systems^34^,^35^,^36^. Typically of order 1 mT, these local *B*_hyp_ fields serve to screen the effect of *B*_0_, causing a Gaussian distribution of fields, *G*(*B*_0_, *B*_hyp_), to be experienced by the ensemble of paramagnetic moments being probed by spin resonance. This field distribution directly translates to Gaussian disorder in the Larmor frequencies of the ensemble, 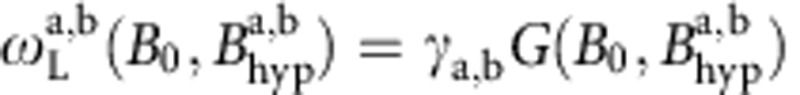 (ref. 10) which, in turn, broadens the range of Rabi frequencies observed under detuning off-resonance^37^ (see further discussion in Supplementary Note 2). This disorder has masked the effect of detuning in previous pEDMR studies such as of poly[2-methoxy-5-(2′-ethylhexyloxy)-p-phenylene vinylene] (MEH-PPV)^27^, which is illustrated in Supplementary Fig. 1. In contrast, the π-conjugated thiophene chains in PEDOT are heavily p-doped to support hole transport and are stabilized by ions in the PSS, which itself does not contribute to charge transport^33^. The ratio of the hydrogen fraction between monomers of PEDOT and MEH-PPV is 1:12, with the actual hyperfine field strength highly dependent on the molecular geometry and the electron wavefunction^38^. The conductivity of PEDOT:PSS thin films drops strongly from room temperature to 5 K (refs 39, 40), where it exhibits semiconductor characteristics^41^. Holes are localized to PEDOT domains within the PSS matrix due to limitations in thermally activated hopping transport^33^,^40^,^42^. Figure 2a compares the pEDMR resonance linewidths of MEH-PPV and PEDOT:PSS at 5 K. The wider distribution present in MEH-PPV is due to significant hyperfine broadening of order 1 mT (refs 8, 10, 21, 43). The PEDOT:PSS spectrum, on the other hand, is nearly three times narrower, indicating a reduction in *B*_hyp_ by at least the same factor. Note that without measuring the resonance linewidth as a function of both *B*_0_ field and microwave frequency, we cannot differentiate hyperfine broadening from broadening due to a distribution in *g*-factors of the charge-carrier spins, which arises due to spin–orbit coupling^8^. Thus, the hyperfine fields in PEDOT:PSS may be even smaller than what the linewidth in Fig. 2a indicates. Figure 2b establishes that we measure these spectra in a regime not influenced by power broadening from the microwave *B*_1_ field since the linewidth saturates at low *B*_1_. We note that identifying polymer-based semiconductors that exhibit such narrow resonances is critical for magnetometry applications utilizing organic semiconductors, for which magnetic field resolution is proportional to linewidth^8^.

### Fundamental and harmonic Rabi oscillations under detuning

**Figure 3:**
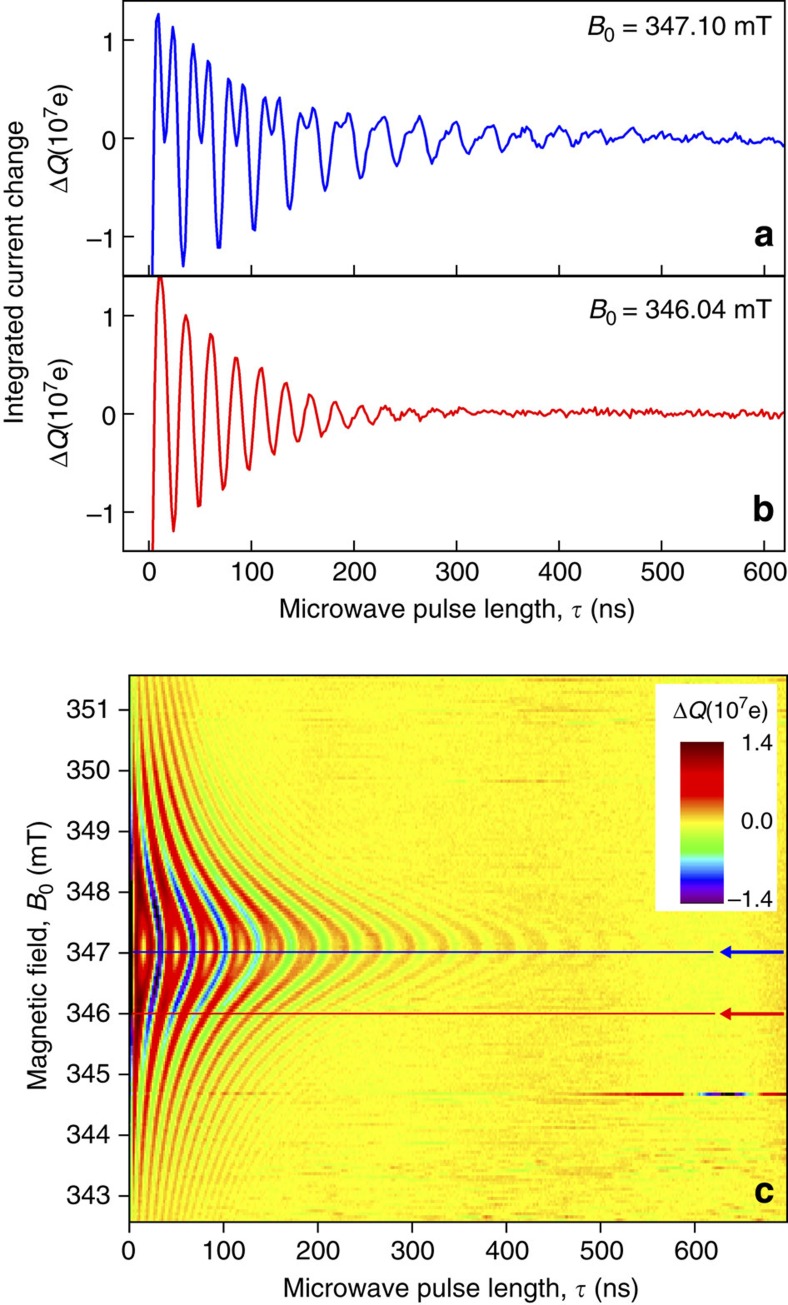
Time evolution of Rabi spin-beat oscillations as a function of *B*_0_-detuning. The data displayed represents the baseline-corrected charge Δ*Q* as a function of the applied pulse length *τ* and the static magnetic field *B*_0_ for a radiation pulse of frequency *f*_MW_=9.73660 GHz and amplitude *B*_1_=1.028±0.004 mT. The data reveal driven Rabi nutation between eigenstates of the spin-pair system through a relative change in the number of charges contributing to the device current as a function of microwave pulse length, *τ*. (**a**) Rabi oscillation for a spin pair on resonance, showing beating close to the second harmonic of the fundamental. The beat oscillation is only observed when the driving field, *B*_1_, is larger than the average difference in Larmor separation of the two carriers in the pair, which is determined primarily by hyperfine interactions. (**b**) Rabi oscillation of the spin pair for significant detuning, where only a single spin is driven by the microwave field. (**c**) Oscillations as a continuous function of detuning showing the corresponding change in spin beating and the shift in Rabi frequency. Blue and red arrows indicate the cross-sections shown in **a** and **b**, respectively.

**Figure 4:**
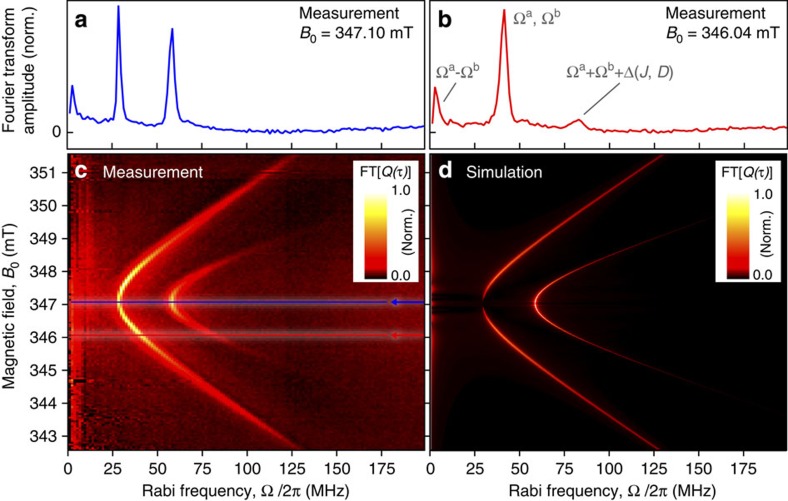
Rabi spin-beat oscillations as a function of *B*_0_-detuning in the frequency domain. (**a**) Rabi frequency components in the on-resonance raw data of Fig. 3a obtained from the Fourier transform of the total charge *Q* (that is, not the baseline-corrected data Δ*Q* displayed in Fig. 3). The harmonic is about twice the fundamental frequency, confirming the presence of a strongly driven pair process, where both spins are resonant in the *B*_1_ field. A frequency peak due to the difference-beat oscillation is also observed close to the origin. (**b**) Frequency components under detuning off resonance (obtained from the raw data *Q*). Fundamental and harmonic shift to higher frequencies under detuning, with the intensity of the harmonic diminishing as only one carrier remains strongly driven. (**c**) The relative change in frequency and amplitude for continuous detuning. (**d**) Simulation based on the stochastic Liouville formalism, utilizing the radical pair spin Hamiltonian given in Supplementary Note 1.

pEDMR reveals the perturbation of the device current following resonant microwave excitation. The device current is governed by a multi-rate transient as described in the Methods, which arises from spin-dependent carrier-pair dissociation and recombination^18^,^44^. Figure 2c shows an example of such a transient of current change from the steady state. The initial enhancement of the current over the first 25 μs is followed by a longer-term (∼600 μs) quenching, which reflects the return to steady state after a resonant population transfer between singlet and triplet eigenstates^18^,^31^. Observation of coherent state manipulation requires time integration over the shaded area, giving the total charge, *Q*(*τ*), involved in the resonant transition during the driving time *τ*. Measuring *Q* on-resonance while applying microwave fields whose *B*_1_ is in excess of the average Larmor separation, 〈Δ*ω*_L_(*B*_0_, *B*_hyp_)〉 (refs 10, 21, 37), leads to Rabi oscillations at the fundamental and the harmonic frequency as shown in Fig. 3a. Note that Fig. 3 displays a baseline-corrected charge Δ*Q*, that is, a second-order polynomial fit function to the raw data *Q* was subtracted from *Q*. This baseline subtraction was introduced solely for improved visualization of the fine structure in Rabi frequency. Since this correction causes a misrepresentation of the low-frequency contributions of the measured data, the quantitative analysis discussed in the following (Fig. 4) was conducted on the raw data *Q*; the correction procedure and the raw data are given in Supplementary Note 3 and Supplementary Fig. 2. Figure 3a shows that a spin-beat oscillation is maintained for over 20 cycles and 700 ns, indicating that damping of Rabi oscillations in this system is fundamentally restricted by the spin coherence time, *T*_2_, rather than 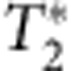. We measured this dephasing time to be 342±2 ns using the spin-echo technique described previously^9^. The presence of both a fundamental Rabi oscillation and a harmonic feature confirms that the species probed is a spin-½ charge pair^29^,^30^,^31^,^32^. In addition, we are able to resolve the spin-beat difference oscillation (at |*ω*_a_−*ω*_b_|) in the Fourier transform, as discussed below (see, for example, the peak close to the origin in Fig. 4a). This difference-beat oscillation is masked in MEH-PPV by the strong hyperfine fields^10^,^27^. The beat oscillation disappears for small *B*_1_ driving fields (data not shown), implying that the pair is weakly exchange coupled^10^,^21^,^25^,^26^. To estimate the relative magnitude of interpair spin coupling, spin beats must be measured while detuning *B*_0_ since the characteristics of off-resonance oscillation frequency components uniquely fingerprint these interactions^28^,^29^,^30^,^32^.

Figure 3b demonstrates the effect of detuning at an off-resonance *B*_0_ field. Off-resonance, a single frequency dominates the oscillation. While the *B*_1_ field is the same as in panel (a), the excitation field no longer drives both carriers. In addition, the Rabi frequency increases, as expected from the analytical expression for a weakly coupled spin pair (equation (1)). The continuous dependence of spin-beat oscillations on detuning is revealed in Fig. 3c by measuring *Q*(*τ*, *B*_0_) above and below resonance.

Quantitative insight into spin–spin coupling comes from Fourier transformation of the time evolution data, making the Rabi frequency components explicit as a function of detuning. Figure 4a–c shows the real part of the Fourier transform *FT*[*Q*(*τ*, *B*_0_], from the raw data *Q* used to plot the baseline-corrected data Δ*Q* in Fig. 3a–c. In Figure 4a, three peaks are seen. The on-resonance fundamental frequency is resolved at 28.9 MHz. The harmonic appears at 58.2 MHz, approximately double the fundamental. This doubling is expected for a strongly driven, weakly coupled spin-½ system, where Ω_R_=*γB*_1_ for the single-spin resonant process, and Ω_R_≈2*γB*_1_ when resonance occurs for both pair partners, as implied by the scheme in Fig. 1b. Since the low-frequency peak in the Fourier transform, arising due to the beating at the Rabi frequency difference (|*ω*_a_−*ω*_b_|), is limited in resolution by the bandwidth of the experiment, we do not discuss this peak further. Fundamental and harmonic components increase in frequency with detuning off-resonance, as exemplified in panel (b). The full detuning dependency is shown in panel (c); two hyperbolas are resolved, corresponding to the detuning of fundamental and harmonic oscillations. The slope of the higher-harmonic hyperbola is approximately twice that of the fundamental, as expected from summing equation (1) over the two carriers.

### Measuring spin-interaction energies with Rabi detuning

**Figure 5:**
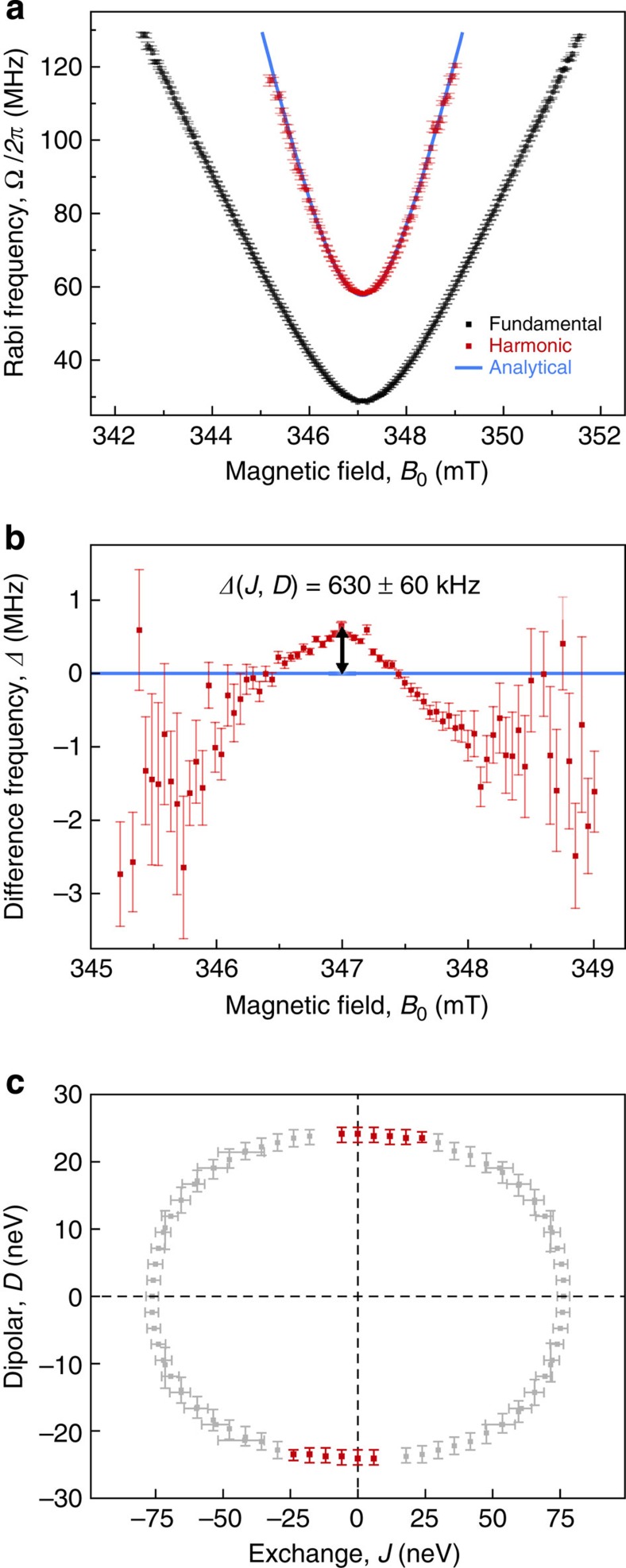
Deviations of measured spin-pair nutation harmonic frequency from the analytical description allowing for intrapair spin–spin interaction strength and intercharge separation to be quantified. (**a**) Frequency peaks for the detuning dependency of the fundamental (black) and harmonic (red) frequency components, plotted in Fig. 4c. The blue line shows the analytical solution for the Rabi frequency of a driven pair of weakly coupled spin-½ carriers (that is, for negligible zero-field splitting), given their measured Larmor separation and the known driving field *B*_1_ as parameters (see refs 29, 31, 32). (**b**) Difference, *Δ*, between measured and computed analytical harmonic frequency as a function of detuning. Since on-resonance, *Δ*, is a function of exchange *J* and dipolar coupling strength *D*, it allows for a direct determination of the intrapair spin–spin interaction strength. (**c**) Numerical simulation of intrapair spin–spin interaction strength. Plot of possible combinations of *J* and *D* that give rise to a shift of the harmonic oscillation frequency in agreement with the measured value *Δ*. By further simulating the detuning behaviour of all frequency components for each of these combinations and eliminating cases that strongly diverge from observation (grey), bounds are placed on the magnitudes of *J* and *D* (red), yielding |*J*|<30 neV and |*D*|=23.5±1.5 neV (see associated discussion in Supplementary Notes 5 and 6). Error bars in the simulation arise from the experimental uncertainty in *Δ* of 60 kHz. Vertical error bars denote simulations where *J* is fixed and *D* is varied, and vice versa for horizontal error bars.

We simulate the detuning behaviour under the given experimental conditions by employing a stochastic Liouville formalism^28^,^30^,^31^,^32^,^37^ (see Methods). The agreement between measurement and simulation, shown in Fig. 4d, is striking, with the primary difference being that the measured data have a reduced frequency resolution due to the finite coherence time of the spin system probed. The specific detuning behaviour allows us to constrain the zero-field splitting parameters *J* and *D* since these directly control the Rabi frequency components on detuning^28^,^29^,^30^,^31^ (see, for example, Fig. 5c). Note that hyperfine interactions can be neglected in the spin Hamiltonian used for this simulation since exchange and dipolar interactions are properties of the mutually coupled pair irrespective of the individual hyperfine field experienced by the pair partners. In addition, the spin–orbit interaction is implicitly accounted for by using the measured difference in *g*-factor for the pair (see Supplementary Note 4 and Supplementary Fig. 3), which enters the spin Hamiltonian of the pair system as a Larmor separation as indicated in Fig. 1c (refs 30, 32).

The effect of small yet finite *J* and *D* in this system can be understood by comparison with the analytical theory for spin-dependent rates controlled by Rabi frequency detuning in the weak coupling limit (see refs 29, 31, 32), as shown in Fig. 5a. The frequency components at each *B*_0_ (that is, the peaks in Fig. 4a,b) are extracted with Lorentzian fits since the frequency resolution of the Fourier transform is below the natural linewidth^45^. Black and red data points give Ω_R_ for the fundamental and harmonic oscillation frequency, respectively. The blue line shows the result of computing the analytical function, equation (1), for the harmonic, using only measured parameters and no free variables^29^,^31^,^32^. Fig. 5b shows the difference *Δ* between measured and analytically obtained harmonic frequencies. This difference of *Δ*=630±60 kHz is determined by the fine structure, the result of non-negligible spin–spin interactions within the pairs. In the absence of an analytical expression for *Δ*(*J*, *D*) to compare this observation to, we take the stochastic Liouville formalism to numerically model the system. By focusing on the harmonic frequency shift for a large set of combinations of *J* and *D* values for the on-resonance case, the subset of combinations of *J* and *D* values is determined, which matches the experimental data. Figure 5c shows the results for this systematic variation of *J* and *D* energies, indicating only those combinations that lead to the experimentally observed *Δ*. Although the harmonic undergoes a positive frequency shift for all relative sign combinations of *J* and *D*, its dependence on these parameters is non-linear for small values of *J* and *D* (see Supplementary Note 1 and Supplementary Fig. 4 for further discussion).

This analysis only considers the combinations of *J* and *D* that give rise to the observed shift in harmonic frequency, but spin–spin interaction also affects the fundamental Rabi frequency. Much stricter bounds on the magnitudes of *J* and *D* are found by considering the detuning behaviour of all frequency components for each of the combinations shown in Fig. 5c and eliminating cases which exhibit strong divergence (see Supplementary Note 6 and Supplementary Figs 5–7). The combinations of *J* and *D* in panel (c) that are eliminated in this way have been greyed out, while those that reproduce all experimentally observed frequency components are marked in red. The resulting values of exchange and dipolar interaction strengths are then narrowed to |*J*|<30 neV and |*D*|=23.5±1.5 neV. The total spin–spin interaction energy, |*J*|+|*D*|, within the spin-pair then yields a magnetic field correction of order 200 μT, depending on the limit of the exchange. As a consequence of the low error in measuring *D*, the average intrapair separation distance is calculated^20^ to be 2.1±0.1 nm (see Supplementary Note 7). This value constitutes the average spacing between two carriers whose spin wavefunction remains quantum mechanically entangled so as to lift the degeneracy between singlet and triplet pair states. The existence of such coherent interactions between distinct carriers for a pair and the resulting entanglement, which potentially occurs between intermolecular species, has previously been speculated on following measurements of photoinduced charge transfer in bulk heterojunctions using the transient Stark effect^46^,^47^.

## Discussion

PEDOT:PSS, although an unlikely candidate owing to its room-temperature conductivity^39^, has proven to be the best-suited organic conductor material system as yet for exploring the intricate coherence phenomena affecting spin-dependent reactions in molecular systems. As the detailed behaviour of these coherence phenomena is fundamentally governed by the magnitude and type of spin–spin interactions, we are able to use detuning in the spin-pair Rabi oscillation as a spectroscopic method of quantifying the spin interaction energy as well as carrier-pair separation. We stress that these results are indifferent to the actual charge state of the carrier-pair system (that is, bipolar or unipolar). Owing to the heavy oxidation state of the hole-transporting PEDOT and the lack of charge transport within PSS, one may be tempted to assign the pair process observed to unipolar hole-bipolarons. These may conceivably exist in adjacent PEDOT domains separated by an intervening PSS layer that partially screens the Coulombic repulsion between like particles, and could arguably contribute to conductivity in a multi-rate current transient under pEDMR. However, the fact that the PEDOT:PSS pEDMR resonance is entirely blocked by depositing MEH-PPV on top, within an organic light-emitting diode geometry^7^,^10^,^12^,^27^, speaks strongly against a unipolar hole-hole process occurring in the measurements discussed here. Another possibility is that we monitor a spin blockade effect within PEDOT domains, moderated by the ionic stabilization between PEDOT and PSS. This situation would effectively constitute an electron–hole polaron pair. From the dramatic reduction in conductivity at low temperature, however, it is also conceivable that both electron and hole are injected directly into the polythiophene chains, giving the familiar bipolar polaron-pair process. Such a bipolar pair process was recently even observed in neat C_60_ films, although fullerenes are considered, like PEDOT:PSS, to be prototypical unipolar conductors^14^.

The measurement of fine-structure splitting in Rabi flopping presented here constitutes a highly sensitive spectroscopy technique with a rigorous theoretical basis^28^,^29^,^30^,^31^,^32^,^48^ that is equally amenable to observables other than electrical current, such as luminescence^21^,^49^. The comprehensive access to quantitative parameters of the radical pair mechanism offers a powerful methodology for exploring spin-coordinated states of both organic^10^,^21^,^27^ and inorganic^25^,^49^ systems. It is therefore ideal for determining metrics to describe complex phenomenological effects, such as organic magnetoresistance^36^,^50^,^51^. We anticipate that the technique presented here could be particularly helpful in characterizing the spin-dependent transport mechanisms in nominally unipolar materials, which can show magnetoresistance in excess of 2,000% (ref. 34). In addition, this technique is potentially relevant to the spin chemistry of avian magnetoreception^52^,^53^ since its applicability has no fundamental lower magnetic field limit. It could conceivably be used in combination with conventional optical probes of the radical pair mechanism in reaction-yield detected magnetic resonance close to zero external magnetic field^54^.

## Methods

### Device fabrication

Pulsed EDMR samples were designed as thin-film devices of a geometry accommodated by the 5 mm Bruker Flexline pulsed ESR microwave resonator^12^. PEDOT:PSS devices consisted of ITO/PEDOT:PSS/Al layers. The PEDOT:PSS layer was spin coated in ambient conditions at 2,000 r.p.m. This film was then dried on a hot plate set to 200 °C for 5 min. A thermal evaporation unit incorporated into an N_2_ atmosphere glovebox was then used to deposit 150 nm aluminium electrodes under a working vacuum pressure below 10^−6^ mbar. MEH-PPV devices were made of layers of ITO/PEDOT:PSS/MEH-PPV/Ca/Al with PEDOT:PSS and the Al top electrode prepared analogously to the PEDOT:PSS devices. MEH-PPV was spin coated from toluene at 1,500 r.p.m. inside the glovebox. Commercial PEDOT:PSS material was obtained from H.C. Starck (Clevios 650), and the MEH-PPV dry polymer from American Dye Source (ADS100RE). MEH-PPV solutions were made at a 7 mg ml^−1^ concentration by dissolving in toluene.

### pEDMR spectroscopy

Changes in current reveal magnetic resonant modifications of the spin of paramagnetic electron states which impact the free carrier density. Under coherent spin excitation, spin states are altered during the very short applied pulse^12^,^13^. The spin-dependent electronic transition rates whose dynamics take place on timescales in excess of the pulse lengths are, therefore, abruptly changed. Long after the end of the pulse (on micro- to millisecond timescales), the modified spin-dependent rate will eventually return to its natural steady state. This electronic relaxation process takes place exponentially for each spin-pair eigenstate and the macroscopic superposition of the different rates is therefore described by a multi-exponential transient. Gliesche *et al*.^30^ showed that the charge *Q* obtained from a partial integration of this current transient represents the change of the density matrix element 
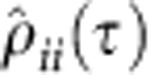
 of the *i*-th eigenstates from its steady state, 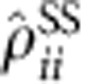, when the integration interval overlaps significantly with the corresponding electronic relaxation transient of this state. The measurement of *Q*(τ) as a function of the pulse length τ therefore reveals the dynamics of 
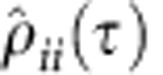
 during coherent excitation^31^. Additional information regarding the technique is available in Supplementary Note 8.

### Experimental set-up

All pEDMR measurements were conducted using a Bruker Elexsys E580 pulsed EPR spectrometer with electrical access to samples within the microwave resonator. Devices were operated at T=5K at a constant bias (∼1.2 V for PEDOT:PSS, ∼12 V for MEH-PPV). During magnetic resonance, the small changes to current were first amplified with an SR-570 low-noise current preamplifier with a bandwidth of 100 Hz–1 MHz. This amplified signal was recorded by a 250 Megasamples s^−1^, 8-bit digitizer within the Elexsys spectrometer (Bruker SpecJet). Custom control sequences programmed into the EPR control software (Xepr) coordinated the microwave pulse scheme, data acquisition and sequential shot averaging. Measurements of resonance linewidth and time dynamics required averaging over 10,240 shots, with a shot repetition period of 510 μs. To prevent any coherent modulation artefacts of the resonance lineshape, linewidth measurements required microwave pulse lengths of 4 μs to measure within the quasi-cw regime. As outlined in Supplementary Note 4 and shown in Supplementary Fig. 3, a double-Gaussian fit of the non-power-broadened resonance lineshape was applied for each point in the time domain, allowing precise measurement of the relative Larmor separation between the two spin-½ carriers (that is, the difference in *g*-factors, Δ*g*=1.53 × 10^−4^±5 × 10^−6^). Note that in spite of the minute *g*-factor difference Δ*g* between pair partners, the pairs are still in the weak coupling regime since random hyperfine field induced differences between the pair partners' Larmor frequencies are significantly above the Δ*g* induced differences as well as the exchange and dipolar coupling (as indicated in Fig. 1c). Coherent measurements of *Q* were performed with minimal microwave attenuation, resulting in *B*_1_=1.028±0.004 mT, and averaged over 1,024 shots. The resolution in microwave pulse length is 2 ns.

### Numerical solutions to the stochastic Liouville equation

The numerical simulation of Fig. 4d was generated under the assumption of negligible exchange and dipolar interaction within the pair, following the superoperator algorithm described by Limes *et al*.^28^ The resonance parameters for the particular simulation of the measured PEDOT:PSS Rabi-nutation signals were obtained from a double-Gaussian fit of the non-power-broadened resonance lineshape shown in Fig. 2a along with an experimentally known *B*_1_ field strength. This same algorithm was used for the simulation of on-resonance Rabi oscillation components in presence of finite exchange and dipolar interactions within the pairs as well as to determine bounds on *J* and *D*. Further details of this analysis are given in Supplementary Notes 5 and 6.

## Author contributions

K.J.v.S. performed electrically detected spin coherence experiments, the measurement analysis and the numerical simulation work. D.L.B. performed the lineshape experiments and measurement analysis. M.E.L., K.J.v.S. and C.B. contributed to the development of the numerical tools. K.J.v.S. and C.B. conceived the concept. J.M.L. contributed to the discussion and guidance of the project. K.J.v.S., C.B. and J.M.L. contributed to the manuscript preparation. C.B. and J.M.L. oversaw the project.

## Additional information

**How to cite this article:** van Schooten, K. J. *et al*. Probing long-range carrier-pair spin–spin interactions in a conjugated polymer by detuning of electrically detected spin beating. *Nat. Commun*. 6:6688 doi: 10.1038/ncomms7688 (2015).

## Supplementary Material

Supplementary Information Supplementary Figures 1-7, Supplementary Notes 1-8 and Supplementary References

## References

[b1] Ritz T., Thalau P., Phillips J. B., Wiltschko R. & Wiltschko W. Resonance effects indicate a radical-pair mechanism for avian magnetic compass. Nature 429, 177–180 (2004) . 15141211 10.1038/nature02534

[b2] Gegear R. J., Casselman A., Waddell S. & Reppert S. M. Cryptochrome mediates light-dependent magnetosensitivity in *Drosophila*. Nature 454, 1014–1018 (2008) . 18641630 10.1038/nature07183 PMC2559964

[b3] Lambert N. . Quantum biology. Nat. Phys. 9, 10–18 (2012) .

[b4] Gauger E. M., Rieper E., Morton J. J. L., Benjamin S. C. & Vedral V. Sustained quantum coherence and entanglement in the avian compass. Phys. Rev. Lett. 106, 040503 (2011) . 21405313 10.1103/PhysRevLett.106.040503

[b5] Schulten K., Staerk H., Weller A., Werner H.-J. & Nickel B. Magnetic field dependence of the geminate recombination of radical ion pairs in polar solvents. Z. Phys. Chem. 10, 371–390 (1976) .

[b6] Maeda K. . Chemical compass model of avian magnetoreception. Nature 453, 387–390 (2008) . 18449197 10.1038/nature06834

[b7] McCamey D. R. . Spin Rabi flopping in the photocurrent of a polymer light-emitting diode. Nat. Mater. 7, 723–728 (2008) . 18711386 10.1038/nmat2252

[b8] Baker W. J. . Robust absolute magnetometry with organic thin-film devices. Nat. Commun. 3, 898 (2012) . 22692541 10.1038/ncomms1895 PMC3621415

[b9] Baker W. J., Keevers T. L., Lupton J. M., McCamey D. R. & Boehme C. Slow hopping and spin dephasing of Coulombically bound polaron pairs in an organic semiconductor at room temperature. Phys. Rev. Lett. 108, 267601 (2012) . 23005015 10.1103/PhysRevLett.108.267601

[b10] McCamey D. R. . Hyperfine-field-mediated spin beating in electrostatically bound charge carrier pairs. Phys. Rev. Lett. 104, 017601 (2010) . 20366393 10.1103/PhysRevLett.104.017601

[b11] Kotler S., Akerman N., Navon N., Glickman Y. & Ozeri R. Measurement of the magnetic interaction between two bound electrons of two separate ions. Nature 509, 376–380 (2014) . 24943952 10.1038/nature13403

[b12] Boehme C. . Pulsed electrically detected magnetic resonance in organic semiconductors. Phys. Status Solidi B 246, 2750–2755 (2009) .

[b13] Lupton J. M., McCamey D. R. & Boehme C. Coherent spin manipulation in molecular semiconductors: getting a handle on organic spintronics. ChemPhysChem 11, 3040–3058 (2010) . 20602409 10.1002/cphc.201000186

[b14] Boehme C. & Lupton J. M. Challenges for organic spintronics. Nat. Nanotechnol. 8, 612–615 (2013) . 24002071 10.1038/nnano.2013.177

[b15] Silva G., Santos L., Faria R. & Graeff C. EDMR of MEH-PPV LEDs. Phys. B 308-310, 1078–1080 (2001) .

[b16] Lee S.-Y., Paik S., McCamey D. R. & Boehme C. Modulation frequency dependence of continuous-wave optically/electrically detected magnetic resonance. Phys. Rev. B 86, 115204 (2012) .

[b17] Chen Y., Liu R., Cai M., Shinar R. & Shinar J. Extremely strong room-temperature transient photocurrent-detected magnetic resonance in organic devices. Phys. Rev. B 86, 235442 (2012) .

[b18] McCamey D. R., Lee S.-Y., Paik S.-Y., Lupton J. M. & Boehme C. Spin-dependent dynamics of polaron pairs in organic semiconductors. Phys. Rev. B 82, 125206 (2010) .

[b19] Suckert M. . Electrically detected double electron–electron resonance: exchange interaction of P donors and P defects at the Si/SiO interface. Mol. Phys. 111, 2690–2695 (2013) .

[b20] Weber A., Schiemann O., Bode B. & Prisner T. F. PELDOR at S- and X-band frequencies and the separation of exchange coupling from dipolar coupling. J. Magn. Reson. 157, 277–285 (2002) . 12323146 10.1006/jmre.2002.2596

[b21] Lee S.-Y. . Tuning hyperfine fields in conjugated polymers for coherent organic spintronics. J. Am. Chem. Soc. 133, 2019–2021 (2011) . 21275069 10.1021/ja108352d

[b22] Boehme C. & McCamey D. R. Nuclear-spin quantum memory poised to take the lead. Science 336, 1239–1240 (2012) . 22679086 10.1126/science.1223439

[b23] McCamey D. R., Van Tol J., Morley G. W. & Boehme C. Electronic spin storage in an electrically readable nuclear spin memory with a lifetime >100 s. Science 330, 1652–1656 (2010) . 21164011 10.1126/science.1197931

[b24] Lee S.-Y. . Readout and control of a single nuclear spin with a metastable electron spin ancilla. Nat. Nanotechnol. 8, 487–492 (2013) . 23793305 10.1038/nnano.2013.104

[b25] Paik S., Lee S.-Y., McCamey D. R. & Boehme C. Electrically detected crystal orientation dependent spin-Rabi beat oscillation of c-Si(111)/SiO_2_ interface states. Phys. Rev. B 84, 235305 (2011) .

[b26] Behrends J. . Bipolaron formation in organic solar cells observed by pulsed electrically detected magnetic resonance. Phys. Rev. Lett. 105, 176601 (2010) . 21231063 10.1103/PhysRevLett.105.176601

[b27] Baker W. J., McCamey D. R., van Schooten K. J., Lupton J. M. & Boehme C. Differentiation between polaron-pair and triplet-exciton polaron spin-dependent mechanisms in organic light-emitting diodes by coherent spin beating. Phys. Rev. B 84, 165205 (2011) .

[b28] Limes M. E. . Numerical study of spin-dependent transition rates within pairs of dipolar and exchange coupled spins with s=1/2 during magnetic resonant excitation. Phys. Rev. B 87, 165204 (2013) .

[b29] Glenn R., Limes M. E., Saam B., Boehme C. & Raikh M. E. Analytical study of spin-dependent transition rates within pairs of dipolar and strongly exchange coupled spins with s=1/2 during magnetic resonant excitation. Phys. Rev. B 87, 165205 (2013) .

[b30] Gliesche A. . Effect of exchange coupling on coherently controlled spin-dependent transition rates. Phys. Rev. B 77, 245206 (2008) .

[b31] Boehme C. & Lips K. Theory of time-domain measurement of spin-dependent recombination with pulsed electrically detected magnetic resonance. Phys. Rev. B 68, 245105 (2003) .

[b32] Rajevac V. . Transport and recombination through weakly coupled localized spin pairs in semiconductors during coherent spin excitation. Phys. Rev. B 74, 245206 (2006) .

[b33] Elschner A., Kirchmeyer S., Lovenich W., Merker U. & Reuter K. PEDOT: Principles and Applications of an Intrinsically Conductive Polymer Taylor & Francis Group (2011) .

[b34] Mahato R. N. . Ultrahigh magnetoresistance at room temperature in molecular wires. Science 341, 257–260 (2013) . 23828887 10.1126/science.1237242

[b35] Roundy R. C., Vardeny Z. V. & Raikh M. E. Organic magnetoresistance near saturation: mesoscopic effects in small devices. Phys. Rev. B 88, 075207 (2013) .

[b36] Nguyen T. D. . Isotope effect in spin response of pi-conjugated polymer films and devices. Nat. Mater. 9, 345–352 (2010) . 20154693 10.1038/nmat2633

[b37] Michel C. . Influence of disorder on electrically and optically detected electron spin nutation. Phys. Rev. B 79, 052201 (2009) .

[b38] Osikowicz W. . Site-specific electronic structure of an oligo-ethylenedioxythiophene derivative probed by resonant photoemission. New J. Phys. 7, 104 (2005) .

[b39] Bubnova O. . Semi-metallic polymers. Nat. Mater. 13, 190–194 (2014) . 24317188 10.1038/nmat3824

[b40] Aleshin A., Williams S. & Heeger A. Transport properties of poly(3,4-ethylenedioxythiophene)/poly(styrenesulfonate). Synthetic Met 94, 173–177 (1998) .

[b41] Long Y. Z., Duvail J. L., Chen Z. J., Jin A. Z. & Gu C. Z. Electrical properties of isolated poly(3,4-ethylenedioxythiophene) nanowires prepared by template synthesis. Polym. Adv. Technol. 20, 541–544 (2009) .

[b42] Nardes A., Kemerink M. & Janssen R. Anisotropic hopping conduction in spin-coated PEDOT:PSS thin films. Phys. Rev. B 76, 085208 (2007) .

[b43] Malissa H. . Room-temperature coupling between electrical current and nuclear spins in OLEDs. Science 345, 1487–1490 (2014) . 25237097 10.1126/science.1255624

[b44] Wohlgenannt M., Tandon K., Mazumdar S., Ramasesha S. & Vardeny Z. V. Formation cross-sections of singlet and triplet excitons in pi-conjugated polymers. Nature 409, 494–497 (2001) . 11206541 10.1038/35054025

[b45] Glenn R., Baker W. J., Boehme C. & Raikh M. E. Analytical description of spin-Rabi oscillation controlled electronic transitions rates between weakly coupled pairs of paramagnetic states with S=1/2. Phys. Rev. B 87, 155208 (2013) .

[b46] Bittner E. R. & Silva C. Noise-induced quantum coherence drives photo-carrier generation dynamics at polymeric semiconductor heterojunctions. Nat. Commun. 5, 3119 (2014) . 24477069 10.1038/ncomms4119

[b47] Gélinas S. . Ultrafast long-range charge separation in organic semiconductor photovoltaic diodes. Science 343, 512–516 (2014) . 24336568 10.1126/science.1246249

[b48] Boehme C. & Lips K. Time domain measurement of spin-dependent recombination. Appl. Phys. Lett. 79, 4363–4365 (2001) .

[b49] Van Schooten K. J., Huang J., Talapin D. V., Boehme C. & Lupton J. M. Spin-dependent electronic processes and long-lived spin coherence of deep-level trap sites in CdS nanocrystals. Phys. Rev. B 87, 125412 (2013) .

[b50] Schellekens A. J., Wagemans W., Kersten S. P., Bobbert P. A. & Koopmans B. Microscopic modeling of magnetic-field effects on charge transport in organic semiconductors. Phys. Rev. B 84, 075204 (2011) .

[b51] Harmon N. J. & Flatté M. E. Effects of spin-spin interactions on magnetoresistance in disordered organic semiconductors. Phys. Rev. B 85, 245213 (2012) .

[b52] Efimova O. & Hore P. J. Role of exchange and dipolar interactions in the radical pair model of the avian magnetic compass. Biophys. J. 94, 1565–1574 (2008) . 17981903 10.1529/biophysj.107.119362 PMC2242753

[b53] Ritz T., Adem S. & Schulten K. A model for photoreceptor-based magnetoreception in birds. Biophys. J. 78, 707–718 (2000) . 10653784 10.1016/S0006-3495(00)76629-X PMC1300674

[b54] Rodgers C. T., Henbest K. B., Kukura P., Timmel C. R. & Hore P. J. Low-field optically detected EPR spectroscopy of transient photoinduced radical pairs. J. Phys. Chem. A 109, 5035–5041 (2005) . 16833855 10.1021/jp050765z

