# Supplementary material for: Probing long-range carrier-pair spin–spin interactions in a conjugated polymer by detuning of electrically detected spin beating
Source: Nat Commun. 2015 Apr 14;6:6688. doi: 10.1038/ncomms7688 (PMC4403378; doi:10.1038/ncomms7688)
Supplement: Supplementary Information — Supplementary Figures 1-7, Supplementary Notes 1-8 and Supplementary References [file ncomms7688-s1.pdf]

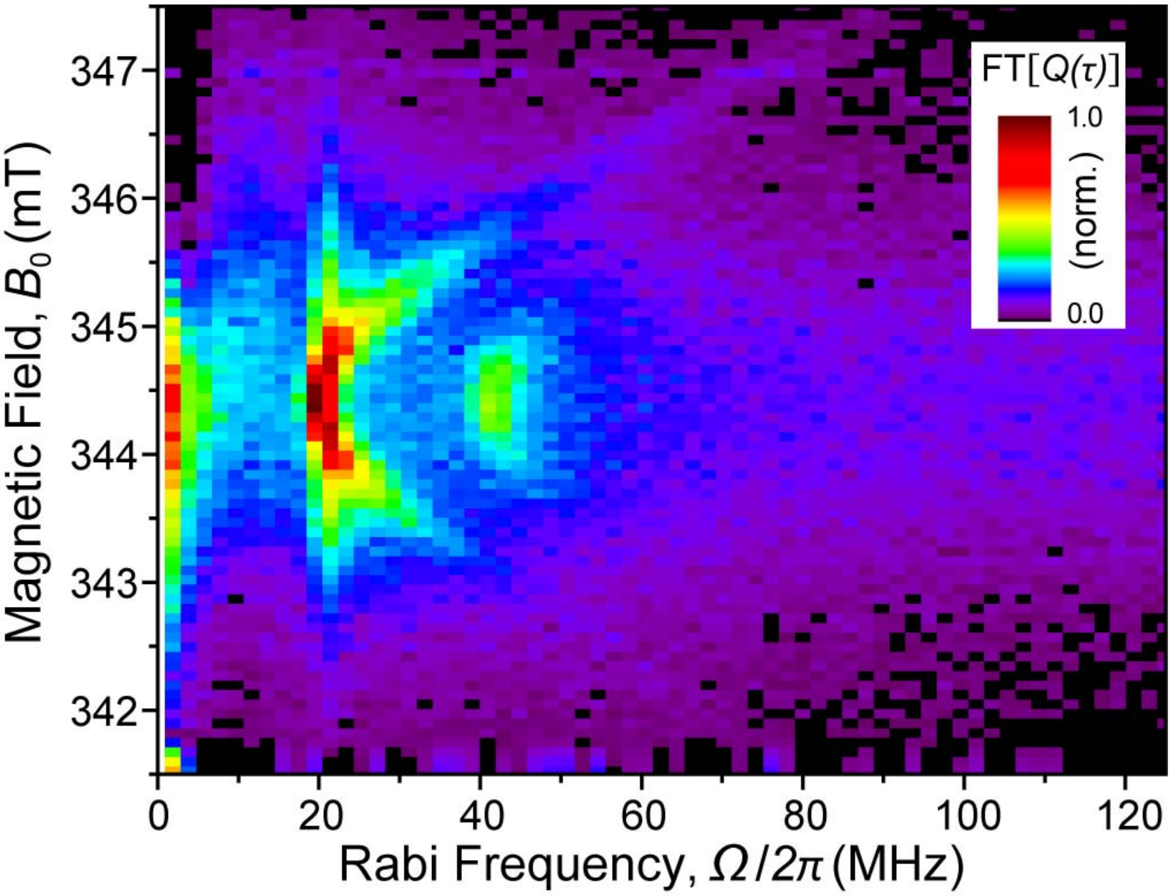

Supplementary Figure 1: Broadening effect of hyperfine fields on detuning of Rabi oscillations. An MEH-PPV OLED is measured in the same manner as the PEDOT:PSS diode considered in the main text, but at room temperature. The spectral features observed display significant broadening in comparison to the PEDOT:PSS signals shown in Figure 4c of the main text. The broadened resonance prevents the clear observation of the behavior of the fundamental Rabi oscillation component under detuning according to Equation (2). No effect of detuning can be observed in the harmonic Rabi oscillation due to the influence of hyperfine broadening. The data were recorded at room temperature on an MEH-PPV diode operated with a steady-state forward current of I_0=100 μA by measuring integrated current change transients after short (a few tens of nanoseconds) pulsed microwave excitation. The OLED device preparation is described elsewhere.[1] In MEH-PPV, very little effect of temperature is observed on coherent spin dynamics.

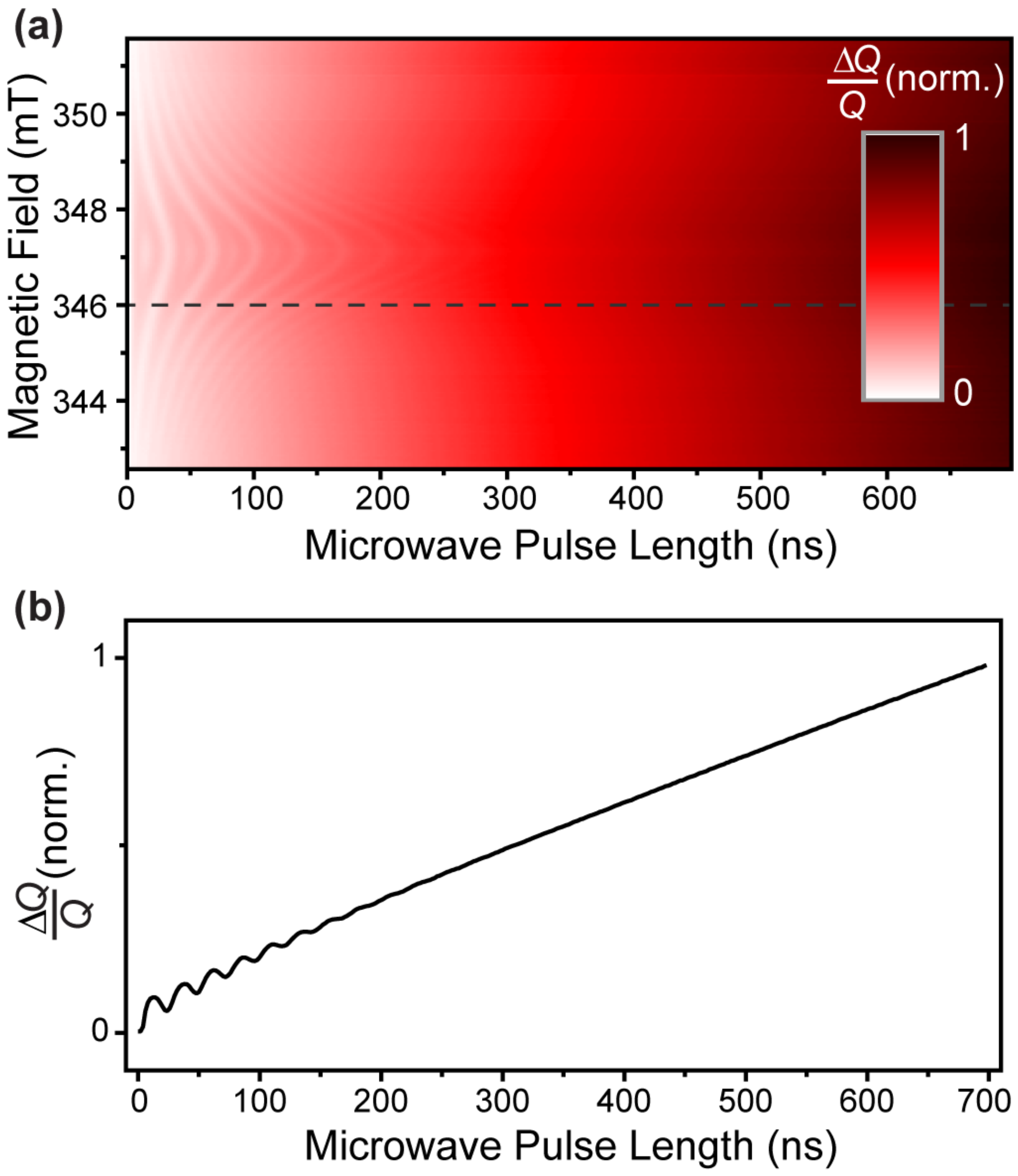

Supplementary Figure 2: Electrically detected Rabi nutations. (**a**,**b**) The same data as shown in the main text Figures 2c and 2b, respectively, but without the subtraction of the baseline function.

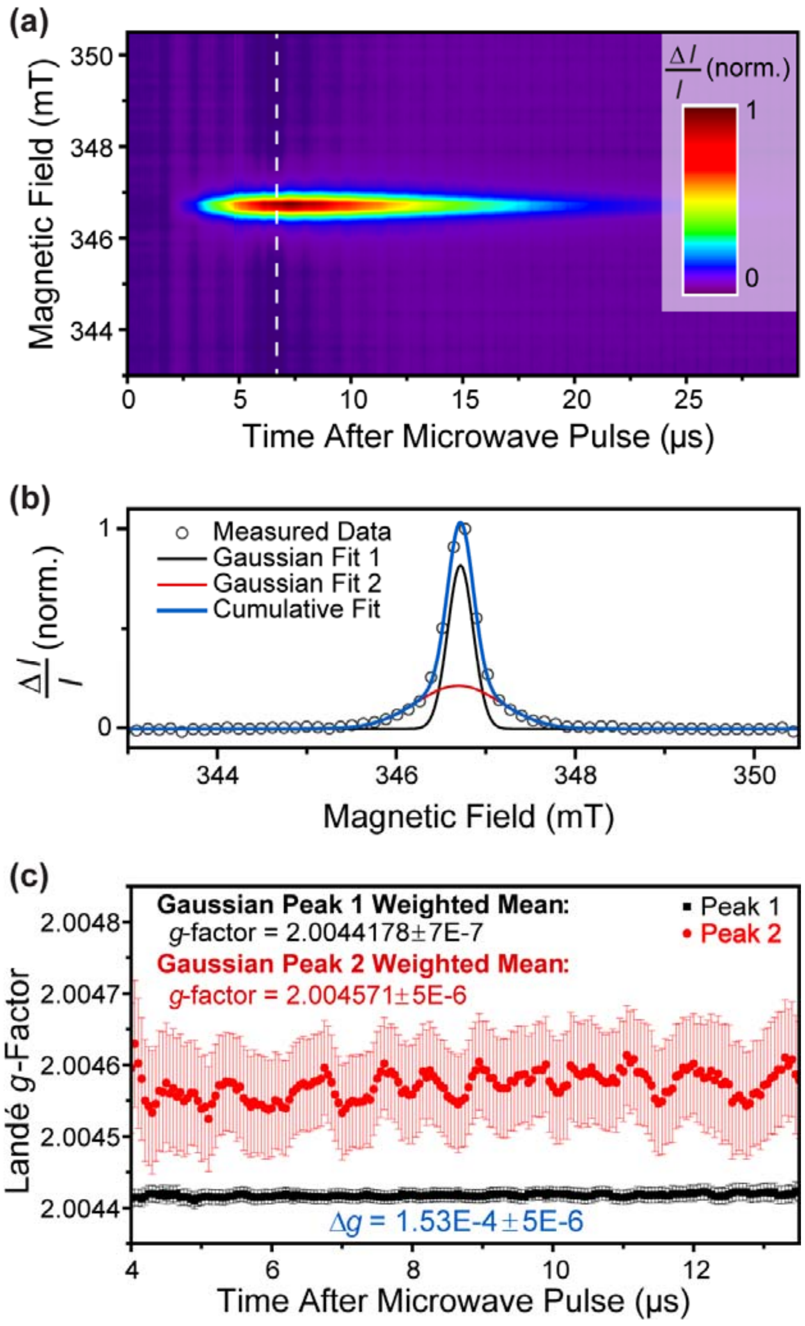

Supplementary Figure 3: Measurement of the Larmor separation within a carrier pair. (**a**) The pEDMR resonance for a PEDOT:PSS diode at 5 K. (**b**) A representative EDMR lineshape, extracted from the resonance in panel (a) at the position of the white dashed line. The spin-pair process results in a double Gaussian profile, which is fit to this data with very good agreement. (**c**) The resonance lineshape at each acquisition time is then independently fit in the same manner, and the Gaussian center positions (*g*-factors) are given as a function of time. Taking the weighted mean of each *g*-factor allows the relative difference, Δ*g*, between them to be determined, which provides a limit to the carrier pair's Larmor separation.

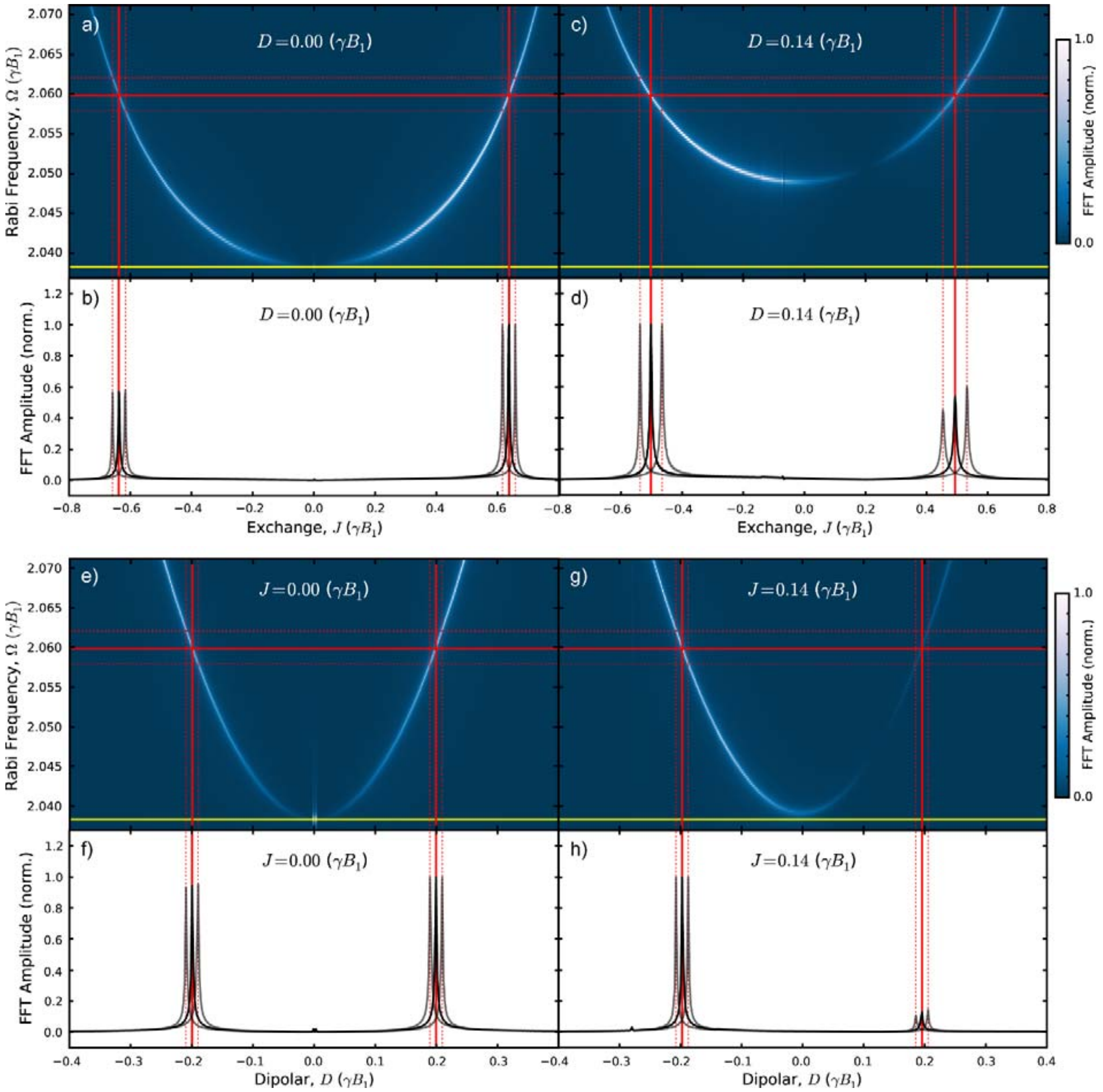

Supplementary Figure 4: Numerical modelling of Rabi harmonic frequency contributions in dependence of the exchange energy *J* and the dipolar coupling strength *D*. (**a**) Dependence as a function of exchange coupling J for $D = 0$. The horizontal yellow line denotes the Rabi beat frequency in the weak-coupling limit, while the solid red horizontal line shows the experimentally obtained shift Δ. Dotted lines correspond to measurement uncertainties. Vertical solid red lines mark the exchange energies which can generate the measured Δ. (**b**) Horizontal cuts of the simulated data in (a) for the experimental value Δ with boundaries of the uncertainty range shown. (**c**,**d**) This simulation is repeated in the presence of finite dipolar coupling. Panels (**e-h**) are similar to (a-d), except for showing the Rabi harmonic frequency shift as a function of dipolar coupling for fixed exchange energies.

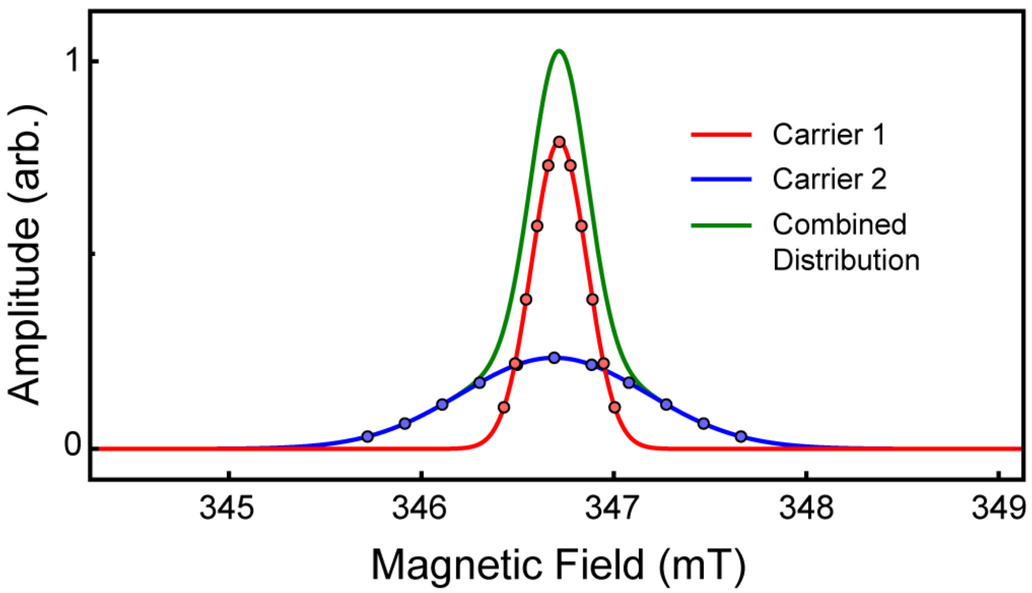

Supplementary Figure 5: Choice of spin pairs between the distribution of each carrier. The pEDMR resonance is composed of an ensemble of spin pairs, with each pair having a different Larmor separation, $\Delta\omega$, and offset from the ensemble average Larmor center. 11 points along each carrier distribution are chosen in order to construct a model of the detuning behavior for the entire ensemble average, exemplified in Supplementary Figure 6. This results in 121 cases of Larmor separation within the double Gaussian distribution.

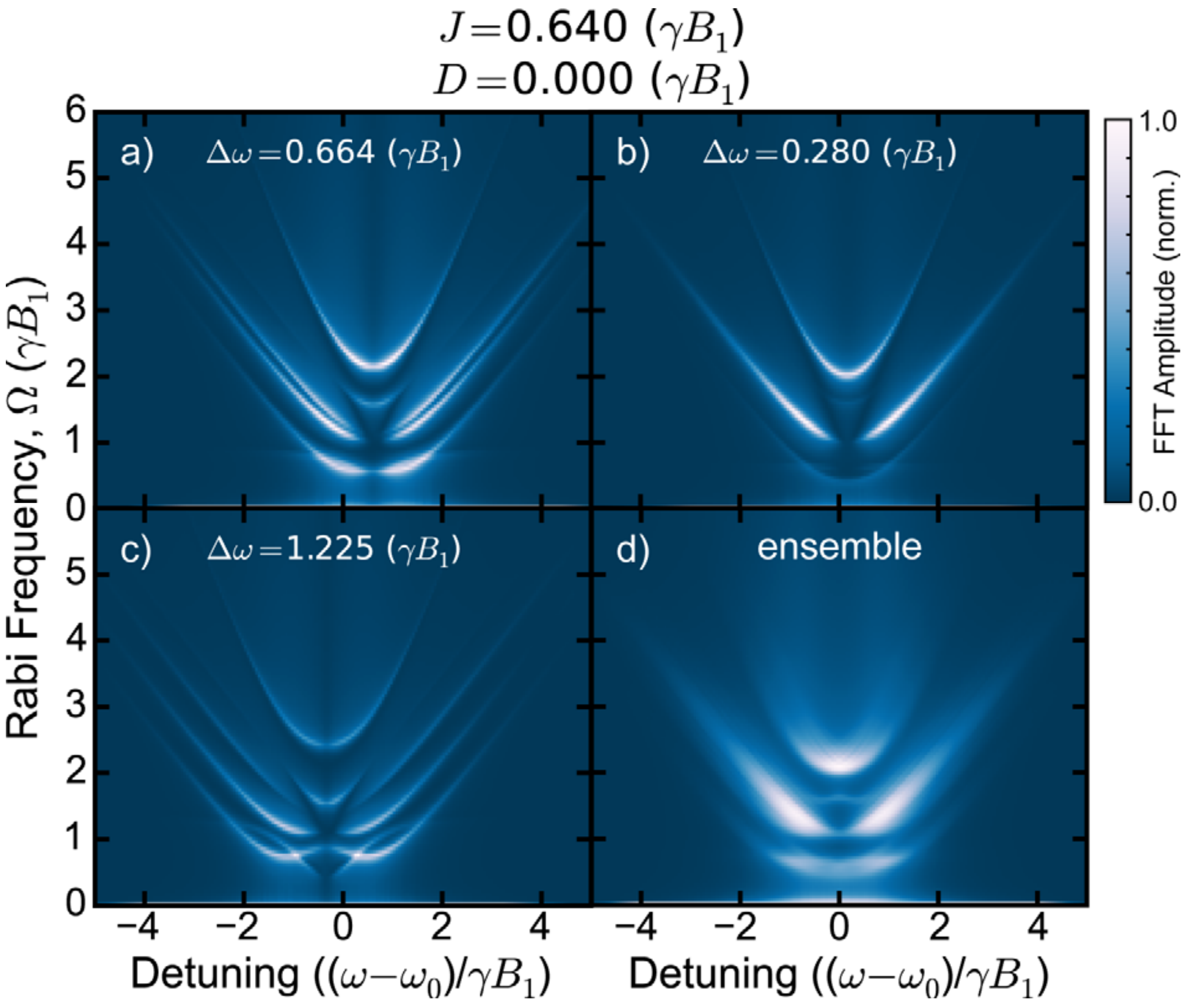

Supplementary Figure 6: Simulation of Rabi frequency detuning of individual spin pairs and their ensemble average in order to place bounds on exchange and dipolar interaction energies. The Rabi frequency detuning for the case of maximal exchange and zero dipolar coupling is simulated for isolated spin pairs within the ensemble distribution. Each spin pair in (**a-c**) is characterized by its Larmor separation, $\Delta\omega$, as labeled. Additionally, the average Larmor center of each pair is shifted along the detuning axis with respect to the ensemble average Larmor center. (**d**) The single spin-pair Rabi frequency detuning behavior was simulated for 121 cases of $\Delta\omega$, weighted according to probability of occurrence in the ensemble, and then added together to construct the ensemble average. The ensemble average has multiple features that are not observed by the experiment, notably the on-resonance frequency components at ~$0.5\gamma B_1$ and ~$1.5\gamma B_1$. This combination of J and D can therefore be excluded as invalid. The same method is used for the inspection of the remaining J and D combinations, allowing strict bounds to be placed on these energies.

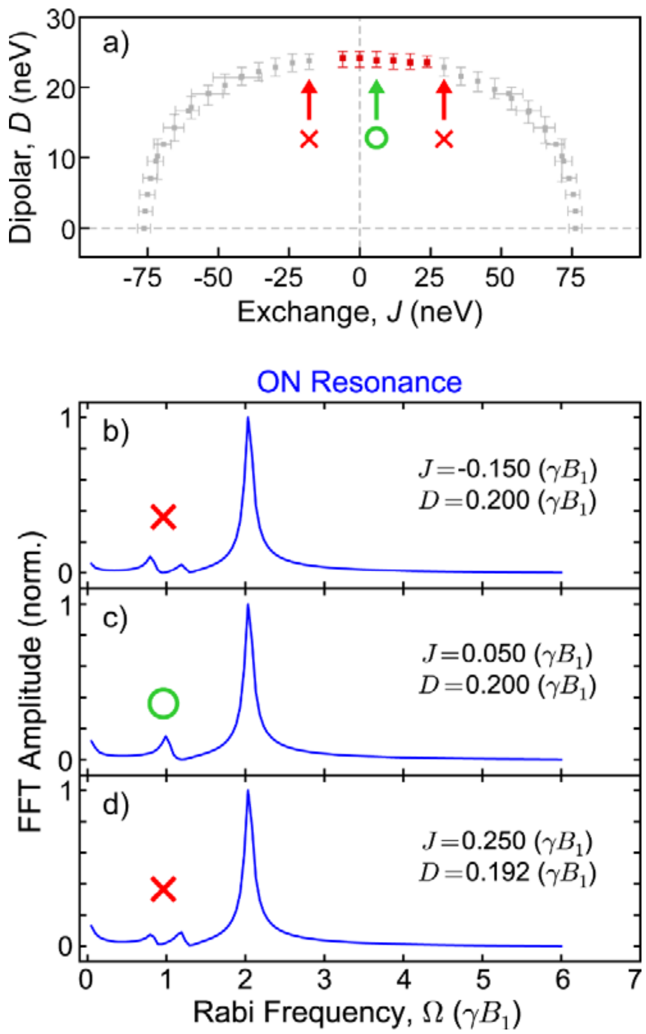

Supplementary Figure 7: Demonstration of the qualitative Rabi-frequency spectra placing bounds on acceptable exchange and dipolar combinations. Bounds on possible J and D combinations which can account for the measured Rabi detuning behavior. (**a**) The combination of spin-spin interaction energy constituents which leads to the experimentally observed offset, Δ, to the harmonic Rabi oscillation is reproduced. The first subsets of combinations that lie outside the range of acceptable values are marked by red arrows and crosses. A valid combination lying directly between these bounds is marked by a green arrow and circle. The corresponding on-resonance Rabi frequency components for each of these combinations is shown in (**b-d**). The combinations that lie outside the bounds of acceptable values (**b**,**d**) display significant splitting in their fundamental frequency component (i.e. around $1\gamma B_1$), which is not observed experimentally. For the case lying within the range of acceptable values (**c**), the distribution of Rabi frequency components matches that of experiment very well. Note that due to the simplifications used for the simulation of the data in (b) through (d), only frequency components but not relative intensities can be directly compared to those observed in the experiment (see discussion in Supplementary Note 6).

Supplementary Note 1.

Spin-Hamiltonian of charge carrier pairs

The spin-Hamiltonian for either unipolar or bipolar charge carrier pairs is represented by an expression

$$\widehat{H} = \hbar\left[\mathbf{B} \cdot \left(\gamma_{\mathrm{a}}\hat{\mathbf{S}}_{\mathrm{a}} + \gamma_{\mathrm{b}}\hat{\mathbf{S}}_{\mathrm{b}}\right) - J\hat{\mathbf{S}}_{\mathrm{a}} \cdot \hat{\mathbf{S}}_{\mathrm{b}} - D\left(3\hat{S}_{\mathrm{a}}^{z}\hat{S}_{\mathrm{b}}^{z} - \hat{\mathbf{S}}_{\mathrm{a}} \cdot \hat{\mathbf{S}}_{\mathrm{b}}\right)\right], \quad (1)$$

where $\gamma_{\mathrm{a,b}}$ is the gyromagnetic ratio and $\hat{\mathbf{S}}_{\mathrm{a,b}}$ is the spin operator for each charge, a and b, of the weakly coupled pair.[2] The first term describes each charge's Zeeman interaction, while the second one describes the isotropic exchange interaction between charges. The third determines the mutual dipolar coupling in the high-field limit, which applies to the conditions discussed here. Since this expression is independent of the charge polarities and $\gamma\hat{\mathbf{S}}$ is a material parameter, the entire spectrum of two-charge spin-½ interactions can be described by simply varying the magnitude of exchange, *J*, and dipolar, *D*, coupling strengths.[3,4] The four resulting energy eigenstates explicitly depend on *J* and *D* (e.g. one singlet and three triplet states for strongly bound excitons). As the eigenbasis states are defined, in part, by the magnitudes of *J* and *D*, the general expression for Rabi nutation frequency between eigenstates of the system is highly sensitive to these spin-coupling parameters.

Supplementary Note 2.

Fine structure of detuning of Rabi oscillations in the presence of large hyperfine coupling

Small inhomogeneous broadening of the charge carriers' magnetic resonance line is crucial for resolving fine-structure in the detuned Rabi spectrum. Since hydrogen is an all abundant constituent of organic semiconductors, hyperfine fields at polaron sites have been found to exceed 1 mT.[1,5] Since these local fields are randomly distributed, they add Gaussian distributed random magnetic fields onto the externally applied magnetic field, resulting in a distribution of Larmor frequencies for charges within the spin pairs. As the detuning term of Rabi's frequency formula depends on the Larmor frequency,

$$\Omega_{\mathrm{R}}^{a,b}(B_0) = \sqrt{\left(\gamma_{\mathrm{a,b}} B_1\right)^2 + \left(\omega_{\mathrm{MW}} - \gamma_{\mathrm{a,b}} \left|\mathbf{B}_0 + \mathbf{B}_{\mathrm{hyp}}^{\mathrm{a,b}}\right|\right)^2}, \qquad (2)$$

Rabi frequencies are distributed when Larmor frequencies are distributed. Therefore, an unambiguous verification of fine structure within the detuned Rabi nutation signal with many common semiconducting polymers, such as MEH-PPV, is difficult due to the ubiquitous hyperfine interactions. Supplementary Figure 1 demonstrates the broadening observed under such conditions for an MEH-PPV OLED at room temperature. Note that the pEDMR linewidth of MEH-PPV does not change appreciably upon cooling to cryogenic temperatures. MEH-PPV therefore does not exhibit a hyperfine coupling regime appropriate for the observation of the fine structure of polaron pairs through detuning of electrically detected spin-Rabi beat oscillations.

Supplementary Note 3.

Electrically detected Rabi nutation

The raw data (device current change) corresponding to the microwave pulse length dependence as a function of magnetic field displayed in Figure 3c of the main text is shown in Supplementary Figure 2. The difference between the two plots is that the main text Figure 3 represents the raw data with an exponential decay function subtracted, which was fitted to the raw data for long pulse lengths. Since this procedure only introduces low-frequency harmonics to the data sets, it allows for a clearer visualization of the Rabi nutation in the main text Figure 3 without distorting the measured Rabi oscillation features around the fundamental and harmonic frequency components. However, as this procedure does distort the measured low-frequency oscillation components, the Fourier analysis displayed in the main text Figure 4a-c was therefore obtained from the raw data without this subtraction.

Supplementary Note 4.

Extraction of Larmor separation from the EDMR spin-pair resonance

In the experiments reported, a (nanosecond-range) pulsed magnetic resonant microwave excitation induces a sudden transition between spin eigenstates, which subsequently changes the device current from a steady state to a value that represents the changed singlet-to-triplet ratio of the pair population. After this abrupt change, a current transient which brings the current back to the steady state can be observed over hundreds of microseconds in the case of PEDOT:PSS diodes at 5 K (Supplementary Figure 3a). The dependence of the initial current change that precedes this transient as a function of the magnetic field reveals the EDMR line. As is characteristic for charge carrier pairs, the EDMR lineshape observed here is a convolution of two Gaussian peaks (Supplementary Figure 3b), corresponding to the pair's individual partner resonances. Note that during the time after a pulse excitation, over which the current change from the steady state returns to zero, the magnitude of the current change will evolve continuously. However, for any time t after the pulse, the dependence of the current change on the magnetic field will resemble the same functions, i.e. it will be described by the same resonance lines. Since the g-factor of each resonant carrier is fixed as a material parameter, the center position of each Gaussian profile also remains fixed (as does the width). In this case, the resonance profile obtained at each time step in the data acquisition can be extracted and fit individually with the two-Gaussian model. Supplementary Figure 3c shows the resulting g-factors obtained through this fitting process as a function of time following microwave excitation of the spin-system. Since each step in time constitutes an independent measurement of resonance lineshape, the weighted mean and associated error for each g-factor can be determined to very high precision. Even though the *absolute* g-factor for each carrier is resolution limited (to ±0.001) by calibration of the Hall sensor used to set the static magnetic field B_0, the *relative* difference between them, Δg, retains the precision of the weighted mean. With each carrier of the pair carrying spin-½, the only contribution to differences in Larmor frequency derives from Δg.

Supplementary Note 5

Calculating exchange and dipolar contributions to the Rabi harmonic frequency shift

The oscillation components caused by a magnetic resonantly driven two spin-½ system (the Rabi-oscillation of the two pair constituents as well as the beat components) are highly sensitive to the magnitudes of intra-pair exchange and dipolar coupling. In the weak-coupling limit ($J = D = 0$), all harmonic components can be analytically solved. In this case, the Rabi beat oscillation component (the harmonic) is fixed to a known frequency since it is the result of a simple summation of fundamental frequencies. As exchange and dipolar energies become non-negligible, this component shifts by a frequency $\Delta(J, D)$, which itself depends on J and D. While general analytical expressions for $\Delta(J, D)$ are not found in the literature, they do exist for particular extremal limits of J and D.[4] Thus, analyzing pair systems in which J and D are arbitrary requires numerical simulations.

In order to explore the effect of J and D on the experimentally observed values of Δ reported in this study, we build upon the stochastic Liouville formalism that was employed in Refs. [2–4,6,7] and was used for the simulation of the experimental data shown in Figure 4d of the main text. For the simulation of the influence of J and D on the pEDMR Rabi-oscillation components, we focus on the simulation of on-resonance Rabi frequency components using simulation conditions of the magnetic field where Δ was extracted from experimental data (cf. main text Figure 5b). Specifically, we focus on the narrow band of frequencies around the harmonic ($\Omega \approx 2\gamma B_1$, the product of the gyromagnetic ratio γ and microwave field strength B_1), encompassing the range of values that includes the weak-coupling limit and the experimentally measured value of Δ. While doing so, we then begin to vary the exchange coupling strength for finite values of D, as shown in Supplementary Figure 4a with $D = 0$. The figure shows that for $J = D = 0$, the harmonic frequency lies at the value expected for the weak-coupling limit (the yellow horizontal line). Note that even in the absence of any spin-spin interaction within the pair, this value is above $\Omega \approx 2\gamma B_1$ since the beat component is determined by a driving radiation field that is slightly detuned from both pair partners due to their Larmor separation $\Delta\omega$.[2,6] As the exchange is increased, the harmonic frequency increases non-linearly and finally reaches the experimentally measured Δ shift (the red horizontal line). Energies are given with respect to the single spin-½ carrier Rabi frequency γB_1. The experimental error of Δ is indicated by horizontal dotted red lines, which translates into an uncertainty range for the extrapolated in value of the exchange interaction in the simulation (vertical dotted red lines). Supplementary Figure 4b displays the cuts taken at the experimental value of Δ (black) and the boundaries of its error ranges (grey).

Supplementary Figure 4c,d repeats this process, but for the assumption of a finite level of dipolar coupling ($D = 0.14\ \gamma B_1$). It is noticeable that the harmonic frequency remains shifted above the weak-coupling limit line (yellow) for all combinations of J and D. The non-linear increase of the oscillation frequency is symmetric about $J = 0$, implying that $\Delta(J, D)$ is unaffected by the signs of J and D. This symmetry is reproduced for all simulated combinations of J and D. Therefore, the total spin-spin interaction energies extracted from Δ depend monotonously on $|J|$ and $|D|$. While the signs of J and D do not affect Δ, they do affect the spectral weight of the frequency components (i.e. the Fourier amplitude), as is seen in panel (c) where the signal all but vanishes when $J \approx 2D$. Supplementary Figure 4e,h displays similar simulation data that is based on choosing a fixed value J and then sweeping through the dipolar coupling D. By repeating this analysis for a large set of fixed values of J and D, an accurate contour of allowed combinations is obtained which all produce the experimentally observed shift Δ of the harmonic oscillation component. These allowed combinations of J and D are displayed in Figure 5c of the main text.

Supplementary Note 6.

Constraining contributions of exchange and dipolar interactions to Rabi frequency detuning

While the ellipse of simulated $\Delta(J, D)$ values can be matched to the experimentally observed Rabi higher-harmonic shift, Δ, each individual J and D combination is not necessarily valid for reproducing the full detuning behavior of all Rabi frequency components. Here, we simulate all J and D combinations in the ellipse of $\Delta(J, D)$ values, eliminating combinations which give rise to frequency components (other than the harmonic) that are not experimentally observed.

In order to faithfully reproduce the detuning behavior, the full ensemble of resonantly-driven spin pairs contributing to the signal is considered. As each carrier species in the pair ensemble is Gaussian distributed in Larmor frequency (cf. Supplementary Figure 3b), there exists a large set of possible Larmor separations, $\Delta\omega$, within individual spin pairs. Since the Gaussian distribution for each carrier species is experimentally known, a choice of 11 points within each distribution was chosen (Supplementary Figure 5), resulting in 121 cases of Larmor separation. The 11 points chosen along each Gaussian profile are equally spaced from the distribution center to a range of $\pm 2\sigma$, where σ is the standard deviation. For each J and D combination in Figure 5c of the main text, the Rabi frequency detuning behavior was simulated for all 121 Larmor separations, weighted according to their probability of occurrence, and added together to construct the ensemble average. Supplementary Figure 6 illustrates this process for the case where J is maximal and D is zero. Panel (a) shows the detuning behavior for a single pair having $\Delta\omega = 0.664\ \gamma B_1$. The Larmor center for this pair is offset to the high side of the detuning range. Panels (b) and (c) give additional single-pair examples, where it is seen that the magnitude of $\Delta\omega$ determines the splitting of Rabi frequency components. The Larmor center for the pair only determines the offset along the detuning axis. Panel (d) shows the ensemble average of the 121 cases of $\Delta\omega$ for this combination of J and D. Although most of the fine structure exhibited by the single-pair detuning is averaged out, the primary frequency components are retained, particularly on resonance at zero detuning. By qualitative inspection alone, the ensemble detuning behavior for this combination of J and D strengths produces frequency components which are not observed in the experimental detuning, This combination of spin-spin interaction energies can therefore be eliminated.

All but 12 of the 84 combinations shown in the main text Figure 5c manifest equally disparate Rabi frequency detuning behavior and have been grayed out. Those combinations of J and D whose ensemble spectra reproduce the frequency components and detuning behavior of the measured Rabi frequency detuning are marked in red. Supplementary Figure 7 shows the edge cases for this selection process. In

order to clarify the deviation and agreement between simulated and measured frequency components for the three J and D combinations shown in panel (a), the on-resonance Rabi Fourier transforms are given. The features common to all three cases are the fundamental at $\sim 1\gamma B_1$, the harmonic at $\sim 2\gamma B_1$, and the difference beat frequency at $\sim 0\gamma B_1$. For the cases in panels (b) and (d), the J and D combinations lie just outside the valid range and a splitting of the fundamental is observed, which does not match experimental observation. The case shown in panel (c) lies in the center of the valid range and agrees with experiment very well. Note that for these data sets, only frequency values and not intensity should be compared to the experimental data set since in these simulations an experimentally measured average Larmor difference was used. The effects of ensemble averaging over very large numbers of spin pairs, the finite width and inhomogeneities of the excitation field, as well as non-rectangular microwave pulses all affect the intensity but not the frequency of the fundamental and are not taken into account. We note that, although the cases shown here lie within the range of positive D, the results are completely symmetric under sign change of J and D.

Supplementary Note 7

Calculation of interaction energy and limit on spin-pair distance

In the weak-coupling limit, the harmonic Rabi oscillation frequency lies at exactly the sum of the two fundamental oscillation frequencies.[2,6] When the harmonic deviates from this sum value, the magnitude of the deviation (main text Figure 5b) provides information on the spin-spin interaction strengths.[3,4] In order to quantify this difference the detuning spectrum must be measured. This spectrum is the Fourier transform $FT[Q(\tau)]$ of the charge $Q(\tau)$ as a function of the resonant pulse length and the magnetic field as shown in Figure 3 of the main text. From this data, the peak frequencies for each of the two oscillation frequencies (fundamental and harmonic) is obtained as a function of the magnetic field B_0. For the experimental data presented, these peaks exhibit a Lorentzian lineshape due to the decay of the observed Rabi oscillations. The intrinsic asymmetric shape of these frequency spectra as described theoretically by Glenn and Raikh[8] could not be observed here as the spectrum is broadened by the rapid decay of the Rabi oscillations. The peak frequencies were determined by individual Lorentzian fits of $FT[Q(\tau)]$ for each magnetic field value (Figure 4a,b of the main text), allowing for a precise determination of frequency peak positions and errors. The results of this analysis are displayed in the main text Figure 5a. The error in determining the fundamental Rabi frequency was found to be 1.4 kHz and arises primarily from uncertainties in the Larmor separation (Δg) between the constituents of the carrier pair due to variations of hyperfine fields. The expected frequency of the harmonic oscillation is also known to the same precision as the fundamental. The deviation, Δ, of the measured harmonic from the value for uncoupled pairs ($J = D = 0$) provides a measure for the spin-spin interaction strength. We find this deviation to be $\Delta = 630 \pm 60$ kHz at zero detuning (shown in Figure 5b of the main text). As described above, knowing this deviation allows us to simulate the range of contributing J and D combinations, resulting in strict bounds for these values ($|J| < 30$ neV and $|D| = 23.5 \pm 1.5$ neV). This finite measure of D allows the average intra-pair separation distance $R_{\mathrm{ab}} = \left(\frac{\mu_0 \mu_{\mathrm{B}}^2 g_{\mathrm{a}} g_{\mathrm{b}}}{4\pi h |D|}\right)^{\frac{1}{3}} = 2.1 \pm 0.1$ nm, to be directly calculated by following reference [9], where μ_0 is the vacuum permeability, μ_{B} is the Bohr magneton, and $g_{\mathrm{a,b}}$ are the g-factors of the paired charges.

As mentioned in the main text, the spin-interaction energy effectively creates a magnetic field correction of the order of $B_{\mathrm{corr}} \approx 200$-$700$ µT, depending on the combination of J and D values used. This correction can be determined in a straightforward manner by considering the relation

$$B_{\mathrm{corr}} = \frac{E_{\mathrm{spin-spin}}}{g_e \mu_{\mathrm{B}}} = \frac{(|J| + |D|)}{g_e \mu_{\mathrm{B}}}. \qquad (3)$$

Supplementary Note 8

Intermediate pair-controlled spin-dependent transitions in organic semiconductors

The spin-dependent currents observed in this study reveal the coherent spin motion of pairs of weakly coupled charge carrier spin states with s=1/2, through recombination rates controlled by the spin pair's permutation symmetry which is determined by spin conservation. This mechanism, called polaron pair recombination, is very similar to spin-dependent radical pair reaction processes that are thought to influence avian magnetoreception. The term "weak" coupling in this context means that dipolar- and exchange interactions are smaller than the average Larmor-frequency differences within the pairs caused by the difference of random hyperfine fields to which the pair partners are exposed. The identification of the pair beating is the characteristic signature which reveals that spin-selection rules apply to pairs of s=1/2 which are subject to Pauli blockade, as discussed previously for polaron-pair recombination in the conjugated polymer MEH-PPV.[1,5]

Spin-dependent recombination through so-called "intermediate pairs" of weakly coupled s=1/2 systems were first described and studied for recombination in inorganic semiconductors by Kaplan, Solomon, and Mott (KSM).[10] Essentially, by describing this mechanism but without explicitly mentioning this fact, KSM recognized that recombining charge carriers can behave in a very similar way to recombining radical pairs in photochemical reactions.

The crucial aspect of the mechanism described by KSM is that the electronic transitions governed by the Pauli blockade take place by initial formation of an intermediate pair state in an arbitrary spin configuration. This pair can either dissociate or undergo an annihilation transition into a pure singlet state (e.g. for recombination into a doubly occupied ground state orbital that annihilates two charge carriers, or through a transport transition which does not annihilate individual charge carriers but removes the intermediate pair). It is this intermediate state which clearly distinguishes the KSM mechanism from other Pauli-blockade mechanisms (e.g. spin-dependent transitions described by Lepin[11]). While KSM originally described the intermediate pair mechanism solely for recombination, concrete implementations of intermediate pair mechanisms can vary greatly in solid-state and molecular systems. These mechanisms exist for spin-dependent transport and recombination, for optical and for dark transitions as long as the pair has an "intermediate" character, which means that it must dissociate first before either pair partner can undergo transitions involving other electrons. Spin-selection rules apply to the pair annihilation transition and are defined by the Pauli-blockade. When spin-orbit coupling is weak enough such that electronic transitions are spin conserved, the projection of the intermediate pair's spin state governs the pair annihilation transition matrix element into a singlet state. Dissociation probabilities of the pairs are

usually spin-independent, yet for spin pair ensembles, this typically nevertheless implies that the dissociation rates depend on the spin state of the dissociating pair as the spin-selection rules that apply to the pair annihilation create an imbalance of singlet and triplet states. Because of this imbalance, a spin-independent dissociation probability will lead to spin-dependent dissociation rates, i.e. even if the pair dissociation process itself is independent of spin (singlet or triplet), the overall dissociation rate will be spin dependent.

Since the work by KSM, the intermediate pair mechanism has been implicated in spin-dependent transport of organic semiconductors in two qualitatively different ways: (i) in the form of "polaron pairs", a term that has been used in the literature exclusively for the description of oppositely charged, bipolar pairs of charge carriers which recombine spin dependently[12]; and (ii) in the form of so-called "bipolarons", weakly spin-spin coupled pairs of *equally* charged carriers whose name pertains to the circumstance that these pairs are precursor states for doubly occupied states, bipolarons.[13] As discussed in the main text, the weakly coupled spin pairs reported for PEDOT:PSS appear to be polaron pairs rather than bipolarons. This conclusion may appear surprising given the fact that PEDOT:PSS is considered a unipolar conductor. However, the pair's charging state is irrelevant for the analysis of spin beating and the conclusions drawn about the measured intra-pair spin-interaction strength since the measurement applies equally to unipolar and bipolar pairs.

Supplementary References

1. McCamey, D. R. *et al.* Hyperfine-field-mediated spin beating in electrostatically bound charge carrier pairs. *Phys. Rev. Lett.* **104,** 017601 (2010).

2. Boehme, C. & Lips, K. Theory of time-domain measurement of spin-dependent recombination with pulsed electrically detected magnetic resonance. *Phys. Rev. B* **68,** 245105 (2003).

3. Gliesche, A. *et al.* Effect of exchange coupling on coherently controlled spin-dependent transition rates. *Phys. Rev. B* **77,** 245206 (2008).

4. Limes, M. E. *et al.* Numerical study of spin-dependent transition rates within pairs of dipolar and exchange coupled spins with s=1/2 during magnetic resonant excitation. *Phys. Rev. B* **87,** 165204 (2013).

5. Lee, S.-Y. *et al.* Tuning hyperfine fields in conjugated polymers for coherent organic spintronics. *J. Am. Chem. Soc.* **133,** 2019–2021 (2011).

6. Rajevac, V. *et al.* Transport and recombination through weakly coupled localized spin pairs in semiconductors during coherent spin excitation. *Phys. Rev. B* **74,** 245206 (2006).

7. Glenn, R., Limes, M. E., Saam, B., Boehme, C. & Raikh, M. E. Analytical study of spin-dependent transition rates within pairs of dipolar and strongly exchange coupled spins with s = 1/2 during magnetic resonant excitation. *Phys. Rev. B* **87,** 165205 (2013).

8. Glenn, R., Baker, W. J., Boehme, C. & Raikh, M. E. Analytical description of spin-Rabi oscillation controlled electronic transitions rates between weakly coupled pairs of paramagnetic states with S=1/2. *Phys. Rev. B* **87,** 155208 (2013).

9. Weber, A., Schiemann, O., Bode, B. & Prisner, T. F. PELDOR at S- and X-Band Frequencies and the Separation of Exchange Coupling from Dipolar Coupling. *J. Magn. Reson.* **157,** 277–285 (2002).

10. Kaplan, D., Solomon, I. & Mott, N. F. Explanation of the Large Spin-Dependent Recombination Effect in Semiconductors. *J. Phys.* **39,** (1978).

11. Lepine, D. J. Spin-Dependent Recombination on Silicon Surface. *Phys. Rev. B* **6,** 436–441 (1972).

12. Frankevich, E. L. *et al.* Polaron-pair generation in poly(phenylene vinylenes). *Phys. Rev. B* **46,** 9320–9324 (1992).

13. Bobbert, P. A., Nguyen, T. D., Van Oost, F. W. A., Koopmans, B. & Wohlgenannt, M. Bipolaron mechanism for organic magnetoresistance. *Phys. Rev. Lett.* **99,** 216801 (2007).
